# Autophagy Activation by Hypoxia Regulates Angiogenesis and Apoptosis in Oxidized Low-Density Lipoprotein-Induced Preeclampsia

**DOI:** 10.3389/fmolb.2021.709751

**Published:** 2021-09-09

**Authors:** Yamei Li, Xueya Zhao, Biwei He, Weibin Wu, Huijuan Zhang, Xingyu Yang, Weiwei Cheng

**Affiliations:** ^1^ International Peace Maternity and Child Health Hospital, Shanghai, China; ^2^ Shagnhai Key Laboratory of Embryo Original Diseases, Shanghai, China

**Keywords:** early-onset preeclampsia, hypoxia, oxidized low-density lipoprotein, autophagy activation, apoptosis

## Abstract

**Objective:** Autophagy influences a wide range of physiological and pathological processes in the human body. In this study, we aimed to investigate the role of autophagy in early-onset preeclampsia (EOPE); autophagy activation by hypoxia could rescue impaired angiogenesis and apoptosis in preeclampsia, leading by ox-LDL.

**Methods:** Transmission electron microscopy was applied to identify autolysosomes in trophoblast cells of the placenta apical region. Quantitative real-time polymerase chain reaction, Western blot, flow cytometry, and wound-healing assays were adopted to determine autophagy activity, angiogenesis, and apoptosis in placenta tissues or HTR8/SVneo cells.

**Results:** Autophagy activity was inhibited in the placenta of women who experienced EOPE; autophagy activation by hypoxia enhanced the migration ability, rescued ox-LDL–mediated impaired angiogenesis in HTR8/SVneo cells [vascular endothelial growth factor A (VEGFA) downregulation and FMS-like tyrosine kinase-1 (FLT1) upregulation], and protected against cell apoptosis (BAX downregulation).

**Conclusion:** Autophagy could maintain the function of trophoblast cells by differentially regulating the expression of VEGFA and FLT1 and protecting against cell apoptosis at the maternal–fetal interface, potentially related to prevention of preeclampsia.

## Introduction

Preeclampsia (PE) is defined as hypertension with a blood pressure (BP) ≥ 140/90 mmHg and substantial proteinuria that is ≥300 mg in 24 h at or after 20 weeks of gestation. The PE with a gestational age less than 34 weeks was the early-onset preeclampsia (EOPE) pregnancies (Brown et al., 2018). PE is a major contributor to global maternal and neonatal morbidity and mortality, present in 2–8% of pregnancies, and always in combination with other pregnancy disorders (Khan et al., 2006). The genuine cause of preeclampsia is heretofore unclear; however, dysregulation of the maternal angiogenesis and trophoblast apoptosis are widely accepted as the main contributions to preeclampsia.

Preeclampsia is considered to be of placental origin and is associated with increased oxidative stress, which could disturb angiogenesis and lead cell apoptosis. Angiogenesis is regulated by a complex cascade of molecular events that include the migration of trophoblasts and by an imbalance between proangiogenic and antiangiogenic factors (Carmeliet and Jain, 2011; Karumanchi, 2016). After implantation, trophoblast cells proliferate and differentiate through two potential pathways described as villous and extravillous: villous cytotrophoblast cells fuse into the multinucleated syncytiotrophoblast, which forms the outer epithelial layer of the chorionic villi and extravillous trophoblast (EVT) cells migrate into the decidua and remodel uterine arteries (Gude et al., 2004) The impaired invasion of EVT cells into uterine spiral arteries could influence angiogenesis in the placenta and disturb the maternal myometrial spiral artery remodeling, promote FLT1 releasing, and induce maternal intravascular systemic inflammatory response and endothelial dysfunction (Steegers et al., 2010). The state of inflammation can further activate and promote the programmed death of trophoblasts, a process called apoptosis (Raguema et al., 2020). Under inflammation, the activation of apoptosis can backfire on the trophoblasts, significantly disrupting cell migration and placental vascularization, while also exacerbating immune responses (Sharp et al., 2010).

During the first trimester of a normal pregnancy, the oxygen tension rises steeply from 20 mmHg (equivalent to 2–3% O_2_) between 8 and 10 weeks of gestation to > 50 mmHg (>6% O_2_) after 12 weeks (Chang et al., 2018). Some studies have demonstrated that autophagy was enhanced under hypoxia at the early stage of the placenta *in vivo* (Chen et al., 2012; Nakashima et al., 2013; Saito and Nakashima, 2014). Autophagy is a basic phenomenon present in eukaryotes, which influences many physiological and pathological processes (Oh and Roh, 2017) and a highly conserved catabolic process that transports cellular proteins and organelles to facilitate the lysosomal degradation pathway (Mizushima, 2007a). There are conflicted statements about autophagy protecting EVT invasion under the physiological hypoxia in the early stages of pregnancy. Some studies demonstrated that high soluble endothelial glycoprotein expression could inhibit EVT invasion and vascular remodeling through the inhibition of autophagy, suggesting that autophagy had physiological functions for maintaining a normal pregnancy (Nakashima et al., 2013; Saito and Nakashima, 2014); Gao suggested that oxidative stress induced excessive autophagy in trophoblast or endothelial cells, affecting cell invasion and placental vasculature and then promoting the development of PE (Gao et al., 2015). However, the mechanism of autophagy influencing preeclampsia is still unclear. Some studies suggested that autophagy may preserve the angiogenic capacity (Abdel-Aziz et al., 2014; Liang et al., 2019) by affecting trophoblast cell homeostasis through maintaining the reactive oxygen species balance after approximately 12 weeks of gestation (Li et al., 2015). Some other studies illustrated that in response to oxidative stress, autophagy could promote survival effects and decrease the induction of apoptosis (Maiuri et al., 2007). Therefore, it is of cardinal significance to explore the effect of autophagy involved in the progress of preeclampsia.

Oxidized low-density lipoprotein (ox-LDL) could cause endothelial dysfunction and is thought to be involved in the initiation and development of hypertension, hyperlipidemia, diabetes, preeclampsia, etc. (Zuniga et al., 2014). Ox-LDL is potentially involved in enhancing oxidative stress, which could downregulate the superoxide dismutase (SOD) expression and was related to the pathogenesis of endothelial dysfunction in PE (Lee et al., 2005; Zuniga et al., 2014). Some studies showed that ox-LDL could inhibit autophagy in macrophages and smooth muscle vascular cells (Li et al., 2014). Autophagy might protect trophoblasts against ox-LDL–mediated inflammation (Zhang et al., 2016). However, ox-LDL–mediated impaired angiogenesis and apoptosis in preeclampsia still need further exploration.

The findings above present us with a rational hypothesis that autophagy activation accompanied by the changes of VEGFA and FLT1 could affect trophoblasts’ migration ability and protect against apoptosis under an ox-LDL–mediated preeclampsia-like condition.

## Materials and Methods

### Placenta Tissue Samples

Placenta tissues were collected from the maternal surface and 2–3 cm away from the root of the central umbilical cord in pregnancies that underwent caesarean delivery. EOPE was defined as follows: a sustained systolic blood pressure ≥140 mmHg or a sustained diastolic blood pressure ≥ 90 mmHg on two independent readings; a total 24-h urine protein ≥300 mg; and the patient’s hypertension and proteinuria having occurred before a gestational age of 34 weeks. Normotensive pregnancy was defined as a pregnancy with normal blood pressure (< 140/90 mmHg) and no proteinuria that was carried to full term. Cases were excluded on grounds of chronic hypertension, chronic kidney disease, diabetes mellitus, heart disease, etc. Placentas were collected from women undergoing caesarean sections at the International Peace Maternal and Child Health Hospital (IPMCH), Shanghai, China. All specimens were collected with the informed consent of the patients. The study was approved by the Research Ethics Committees in the International Peace Maternity and Child Health Hospital, Shanghai, China.

### Transmission Electron Microscopy

The placenta samples were taken from the villous–decidua interface, separated into 1.0-mm^3^ pieces, placed in 2% glutaraldehyde, and rinsed with phosphate-buffered saline (PBS) twice. The samples were then immersed in epoxy resin 618 entrapped liquid with propylene oxide (1:1) for 2 h, epoxy resin 618 entrapped liquid and epoxy propane (2:1) for 12 h, and then embedded in pure epoxy resin with 618 soaking liquid for 6 h at 37°C. TEM (Hitachi, Tokyo, Japan) was used to observe the ultrastructure and autolysosomes of the EVT cells.

### Cell Cultures

The HTR8/SVneo cells were cultured in DMEM (Thermo Fisher, Waltham, MA, United States) with 10% fetal bovine serum (FBS, Transgen Biotech, Beijing, China) at 37°C in a humidified incubator. The cells were randomly divided into seven groups: the Control group, the ox-LDL (Peking Union-Biology, Beijing, China, 100 μg/ml) group, the Hypoxia group, the Hypoxia + ox-LDL group, the ox-LDL + Rapamycin (Rapa, MedChemExpress, New Jersey, United States, 20 nM, 1 h of pre-incubation) group, the Hypoxia + Chloroquine (CQ, MedChemExpress, New Jersey, United States, 40 μM, 12 h of incubation) group, and the Hypoxia + ox-LDL + CQ group. A tri-gas incubator at 37 °C with 5% CO_2_ and 1% O_2_ was used to keep cells under hypoxia. The normoxia environment was at 37 °C with 5% CO_2_ and 20% O_2_.

### Quantitative Real-Time Polymerase Chain Reaction

QRT-PCR was used to detect the mRNA expression of light chain 3 beta (*LC3B*): 5′-CGA​GAG​CAG​CAT​CCA​ACC​AA-3′ (upstream), 5′-CAC​TCA​TGT​TGA​CAT​GGT​CGG-3′ (downstream); *Beclin1*: 5′-GAA​ACC​AGG​AGA​GAC​CCA​GG-3′ (upstream), 5′-CAG​AGT​GAA​GCT​GTT​GGC​AC-3′ (downstream); *VEGFA*: 5′-AGG​GCA​GAA​TCA​TCA​CGA​AGT-3′ (upstream), 5′-AGG​GTC​TCG​ATG​GAT​GGC​A-3′ (downstream); *FLT1*: 5′-GCG​CTT​CAC​CTG​GAC​TGA​CA-3′ (upstream), 5′-GAA​ACT​GGG​CCT​GCT​GAC​ATC-3′ (downstream); *BAX*: 5′-AAG​AAG​CTG​AGC​GAG​TGT​CT-3′ (upstream), 5′-TGG​CAA​AGT​AGA​AAA​GGG​CG-3′ (downstream); *SOD*: 5′-CTC​AGG​AGA​CCA​TTG​CAT-3′ (upstream), 5′-CAG​CTA​GCA​GGA​TAA​CAG​AT-3′ (downstream); and *18s*: 5′-CAG​CCA​CCC​GAG​ATT​GAG​CA-3′ (upstream), 5′-TAG​TAG​CGA​CGG​GCG​GTG​TG-3′ (downstream). After reverse transcription, the QRT-PCR process was performed using a QuantStudio 7 Flex Real-Time PCR System (Thermo Fisher Scientific, MA, United States). The relative mRNA expression of the target gene was calculated using the comparative Ct method with *18s* mRNA expression as the internal control.

### Western Blot Analysis

The placenta tissues and HTR8/SVneo cells were homogenized and lysed in ice-cold radio immunoprecipitation assay (RIPA) lysis buffer (Thermo Fisher Scientific, MA, United States). The lysates were boiled and resolved using SDS-PAGE. SDS-PAGE–separated proteins were transferred onto polyvinylidene difluoride membranes (Bio-Rad, Hercules, United States). Target proteins were probed with indicators following their primary antibodies, which were as follows: anti-LC3B, anti-Beclin1, anti-P62 (Cell Signaling Technology, MA, United States), anti-BAX (Abcam, Cambridge, United Kingdom), anti-GAPDH, anti–β-actin, and anti–β-Tubulin (YEASEN, Shanghai, China). After incubation with a secondary antibody, the bands were captured using an Amersham Imager 600 (GE, MA, United States).

### Immunofluorescence

HTR8/SVneo cells were plated on 24-well plates with a density of 7.5 × 10^4^ cells per well and transfected with a green fluorescent protein microtubule associated protein 1 LC3 (GFPMAP1LC3) adenovirus vector (5 × 10^9^ pfu/mL) (Gene Chemical, Shanghai, China) for 24 h. The virus-transfected HTR8/SVneo cells were named GFPMAP1LC3-HTR8/SVneo with green and red fluorescence. The green fluorescence represented the early stages of autophagy, the red fluorescence represented the advanced stages of autophagy, and the yellow spot (merged with green and red) represented autolysosomes. Artificial counting was used to analyze the number of autolysosomes using a fluorescence microscope. Cells were considered autophagic phages when more than five GFPMAP1LC3 punctas were found in the cytoplasm. The experiments were independently performed at least three times, and we confirmed the validity of the first measurements and then reported only the first measurements.

### Wound-Healing Assay

HTR8/SVneo cells were plated on 6-well plates with a density of 5 × 10^5^ cells per well and allowed to fuse 100% after 24 h. The cell monolayer was scratched using a sterile 10-µL pipette tip and washed thrice with PBS. Then, the serum-free medium was added to eliminate the effect of cell proliferation. Photographs were taken using a microscope at 0 and 24 h. The experiments were independently performed at least three times, and we confirmed the validity of the first measurements and then reported only the first measurements.

### Flow Cytometry

Apoptosis analysis was carried out using an eBioscience™ annexin V-FITC apoptosis detection kit (Thermo Fisher Scientific, MA, United States) and flow cytometry. The cells were harvested and washed with PBS twice, re-suspended in binding buffer, and then stained with annexin V-FITC and PI for 15 min at room temperature. The samples were analyzed by C6 flow cytometry (BD Bioscience, NJ, United States). The apoptosis ratio was calculated using [annexin V (+) PI (−) cells]/total cells×100% (early apoptosis) [annexin V (+) PI (+) cells] /total cells×100% (advanced apoptosis). Three replicates were prepared and analyzed for each group.

### Statistical Analysis

Statistical analysis was performed using GraphPad Prism 7. The results were present as the mean values ± the standard errors. Nonparametric statistical analysis was used to analyze the significance of the differences between the two groups. Comparison of the means for more than two groups was performed using multiple tests. The Holm–Sidak method was used for correcting in multiple comparisons for *post hoc* tests. Counting the number of autolysosomes and analyzing immunostained features were done blindly and applied by repeated measures analysis of variance. The differences were considered significant when *p* was < 0.05.

## Results

### Patients’ Characteristics

The subject characteristics of all patients enrolled in the study are shown in Table 1. The maternal age was similar in the Control (CON) and EOPE groups. The gestational age at delivery was significantly lower in women with EOPE than in women with normal pregnancies. Blood pressure values and proteinuria were significantly higher in women who experienced EOPE than in normal women. The proportion of fetal growth restriction (FGR) in EOPE pregnancies was 20.0%.

**TABLE 1:** Characteristics of the study population.

Characteristic	CON (N = 10)	EOPE (N = 15)	*p* value
Age, year	30.7 ± 1.243	31.5 ± 1.424	0.693
Gestational, week	37.2 ± 0.435	33.6 ± 0.218	< 0.0001
Systolic blood pressure, mmHg	110.0 ± 4.476	143.4 ± 3.534	< 0.0001
Diastolic blood pressure, mmHg	74.5 ± 4.176	97.5 ± 3.264	< 0.0001
Proteinuria, mg/24h	—	2.882 ± 0.473	< 0.0001
Birth weight, g	3,418 ± 104.2	2025 ± 195.5	—
FGR^a^	0 (0.0%)	3 (20.0%)	< 0.0001

Data were shown as the mean ± standard errors.

a FGR was defined as birth weight less than the 10th percentile value for each gestational week at birth in Chinese infants.

### Comparison of Autophagy Levels in Placentas Between Normotensive and EOPE Pregnancies

**FIGURE 1:**
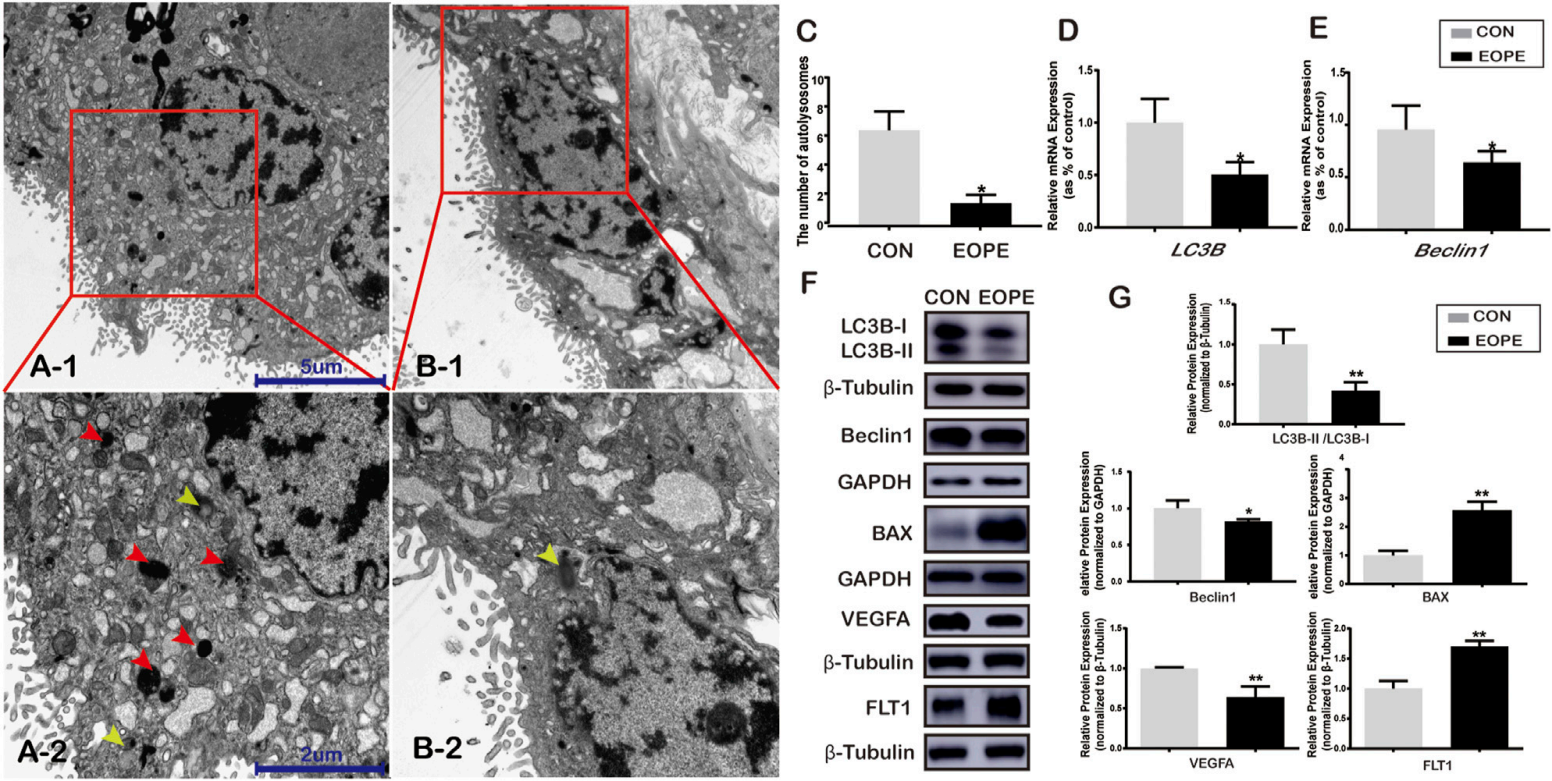
Autophagy and BAX, VEGFA, and FLT1 expression in normotensive and early-onset preeclampsia pregnancies. **(A-1)** Electron micrographs illustrating autolysosomes (red box) in syncytiotrophoblasts from normal pregnancy placentae (CON) (scale bar = 5 μm, 4,000x). **(A-2)** Autolysosomes (red and yellow arrowheads) in syncytiotrophoblasts from EOPE placentae (scale bar = 5 μm, 4,000x). **(B-1)** Autolysosomes (red box) in the syncytiotrophoblast of the CON group (scale bar = 2 μm, 10,000x). **(B-2)** Autolysosome (yellow arrowhead) in the syncytiotrophoblast of the EOPE group (scale bar = 2 μm, 10,000x). **(C)** Number of autolysosomes in the CON and EOPE groups (*N* = 6). **(D)** Relative mRNA expression of LC3B (*N* = 10:15). **(E)** Relative mRNA expression of Beclin1 (*N* = 10:15). **(F)** LC3B-I, LC3B-II, Beclin1, BAX, VEGFA, and FLT1 protein expression and the LC3B-II and LC3B-I ratios in the CON and EOPE groups (*N* = 6). **(G)** Quantification of LC3B-II/LC3B-I, Beclin1, BAX, VEGFA, and FLT1 protein expression normalized to β-Tubulin or GAPDH protein expression in placenta issues (*N* = 6). Data were expressed as normalized ratios (CON = 1). Set average CON = 1, and the other data were compared with it. The results were expressed as means ± standard errors, *p < 0.05, **p < 0.01.

**FIGURE 2:**
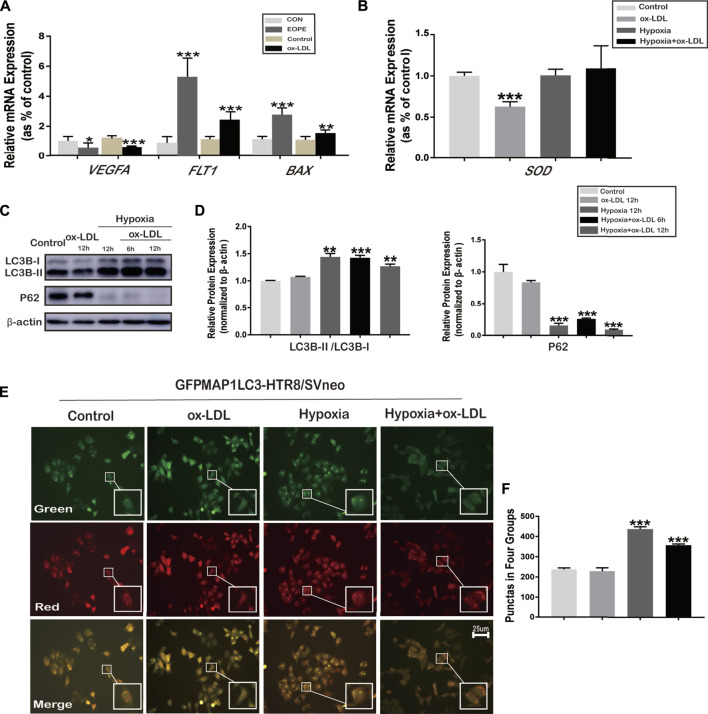
mRNA expression of VEGFA, FLT1, BAX, and SOD, and the phenomenon of autophagy in HTR8/SVneo cells. **(A)** Relative mRNA expression of VEGFA, FLT1, and BAX in placenta tissues (in normal and EOPE women) and in HTR8/SVneo cells treated with ox-LDL for 12 h (*N* = 6). **(B)** Relative mRNA expression levels of SOD in HTR8/SVneo cells treated with hypoxia and/or ox-LDL for 12 h (*N* = 6). **(C)** LC3B-I, LC3B-II, and P62 protein expression and LC3B-II and LC3B-I ratios in HTR8/SVneo cells treated with hypoxia and/or ox-LDL for 6 and 12 h (chloroquine was added for 2 h previously to block the degradation of LC3B). **(D)** Quantification of LC3B-II/LC3B-I; P62 protein expression normalized to β-actin protein expression in HTR8/SVneo cells (*N* = 6). **(E)** Fluorescence microscopy images showing the fluorescence densities of green spots (the early stages of autophagy), red spots (the advanced stages of autophagy), and yellow spots (autolysosomes) in the Control group, ox-LDL group, Hypoxia group, and Hypoxia + ox-LDL group. **(F)** Number of autophagy spots of Figure 2E. Data were expressed as normalized ratios (CON/Control = 1). Set average CON/Control = 1, and the other data were compared with it. Counting the immunostained features to be done blindly and selecting one set of data randomly. The results were presented as means ± standard errors, *p < 0.05, **p < 0.01, ***p < 0.001.

TEM was used to elucidate the autolysosome differences in the syncytiotrophoblast from placentas between women who experienced EOPE and women with normotensive pregnancies. There were shown some autolysosomes, the structures of which contained lysosomal enzymes (Figure 1A-2, red arrowheads) and semi-digested cell contents (Figure 1A-2, B-2, yellow arrowheads). A significant amount of ordered microvilli were observed in the plasma membrane of the syncytiotrophoblast in normotensive placenta tissues, and more autolysosomes were observed in the syncytiotrophoblast apical region from normal placentae in the CON group than in the EOPE group (Figure 1A-1,A-2,C). The microvilli in the EOPE syncytiotrophoblast were irregularly arranged and varied in length and width. In addition, the number of autolysosomes in the EOPE group was relatively reduced compared to that in the CON group (Figure 1B-1, B-2,C).

### Expression of LC3B, Beclin1, BAX, VEGFA, and FLT1 in Placentae

There were significantly decreased mRNA and protein expressions of LC3B (Figure 1D,F,G) and Beclin1 (Figure 1E–G) in women with EOPE compared to those in the CON group. The relative mRNA and protein expression of VEGFA was lower in placentae from the EOPE group than in those from the CON group (Figure1F,G, 2A), while the relative mRNA and protein expression of FLT1 and BAX was significantly higher in the EOPE group than in the CON group (Figure1F,G, 2A).

### Expression of VEGFA, FLT1, BAX, and SOD in HTR8/SVneo Cells

The relative mRNA expression of *VEGFA* was downregulated, whereas that of *FLT1* and *BAX* was upregulated when HTR8/SVneo cells were exposed to 100 μg/ml ox-LDL for 12 h (the ox-LDL group) compared with the untreated group (Control) (Figure 2A). The relative mRNA expression of *SOD* significantly decreased when cells were exposed to 100 μg/ml ox-LDL for 12 h (the ox-LDL group) compared with the untreated cells (the Control group) (Figure 2B). Thus, treatment of 100 μg/ml ox-LDL for 12 h was chosen to stimulate the oxidative stress associated with preeclampsia in HTR8/SVneo cells. No difference was observed in the mRNA expression of *SOD* between the Hypoxia, Hypoxia + ox-LDL, and Control groups over 3 repetitions (Figure 2B).

### Autophagy in HTR8/SVneo Cells Treated With Hypoxia or/and ox-LDL

In order to determine the LC3B expression and experimental conditions, chloroquine was pretreated for 2 h to block the degradation of autophagy. The LC3B-II and LC3B-I ratios were significantly increased in the Hypoxia and Hypoxia + ox-LDL groups, and the protein expression of P62 (known as sequestosome 1, SQSTM1) was reduced, which could interact with LC3B-II and degraded during autophagy (Figure 2C). Fluorescence microscopy images showed that when treated with 100 μg/ml ox-LDL for 12 h, autophagy was not obviously observed in the GFPMAP1LC3-HTR8/SVneo cells (Figure 2E,F, the ox-LDL group). We found that there were strong positive puncta for GFPMAP1LC3 when cells were held in a 1% O_2_ environment for 12 h (Figure 2E,F, Hypoxia and Hypoxia + ox-LDL groups).

### Hypoxia-Induced Autophagy Affected Apoptosis

**FIGURE 3:**
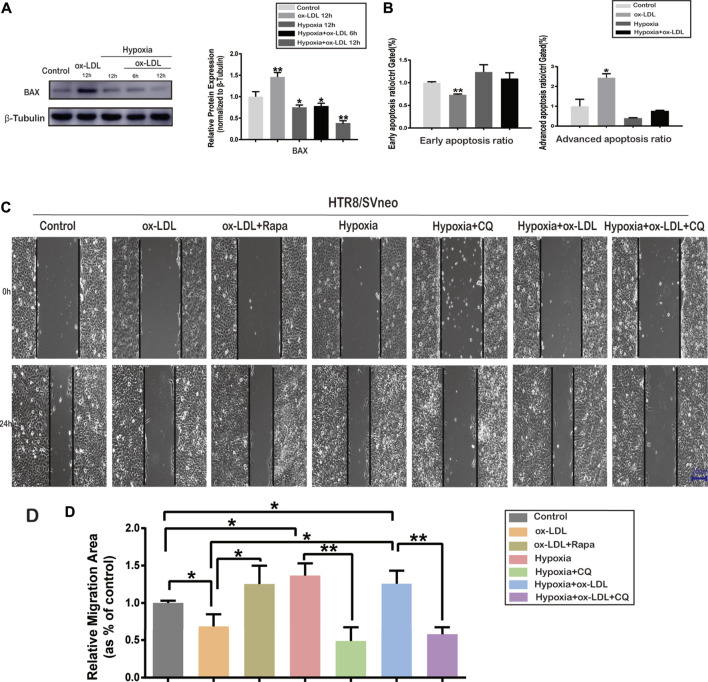
Cell apoptosis ratio and representative images of wound areas in HTR8/SVneo cells*.* **(A)** Protein expression of BAX and β-Tubulin in HTR8/SVneo cells. **(B)** Early and advanced apoptosis ratio in HTR8/SVneo cells. **(C)** Cell scratching images in the Control, ox-LDL, ox-LDL + Rapa, Hypoxia, Hypoxia + CQ, Hypoxia + ox-LDL, and Hypoxia + ox-LDL + CQ groups (scale bar = 25 µm). **(D)** Measurement of the wound area in the seven groups. Data were expressed as normalized ratios (Control = 1). Set average Control = 1, and the other data were compared with it. The results were expressed as means ± standard errors, *p < 0.05, **p < 0.01, ***p < 0.001.

The data showed that the relative protein expression of BAX significantly increased when cells were exposed to ox-LDL (the ox-LDL group) compared with that in the Control group (Figure 3A). When the cells were treated with hypoxia (the Hypoxia group), the protein expression of BAX significantly decreased compared with that in the Control group (Figure 3A). Flow cytometry was used to analyze the apoptosis ratio of HTR8/SVneo cells treated with/without hypoxia and ox-LDL**.** It was found that the early apoptosis ratio was significantly lower in the ox-LDL group than in the Control group (Figure 3B). It was demonstrated that the advanced apoptosis ratio was significantly higher in the ox-LDL group than in the Control group (Figure 3B).

### The Migration Ability of HTR8/SVneo Cells Among the Seven Groups

Wound-healing assay was used to detect the migration ability of HTR8/SVneo cells when treated with hypoxia and/or ox-LDL. The wound area was significantly larger in the ox-LDL group than in the Control group after scratching for 24 h. The wound area was significantly smaller in the ox-LDL combined with Rapamycin group (ox-LDL + Rapa group) than in the ox-LDL group (Figures 3C,D). The wound area was significantly smaller when cells experienced hypoxia (Hypoxia and Hypoxia + ox-LDL groups) than it was when they experienced normoxia (the Control and ox-LDL groups) (Figure 3C,D). The wound area was significantly larger in the case of hypoxia combined with chloroquine (the Hypoxia + CQ group) than it was in the hypoxia group, and the wound area was significantly larger in the Hypoxia + ox-LDL + CQ group than in the Hypoxia + ox-LDL group (Figure 3C,D).

### Effects of Autophagy Activation or Inhibition on the Expression of SOD, LC3B, BAX, VEGFA, and FLT1 in HTR8/SVneo Cells

**FIGURE 4:**
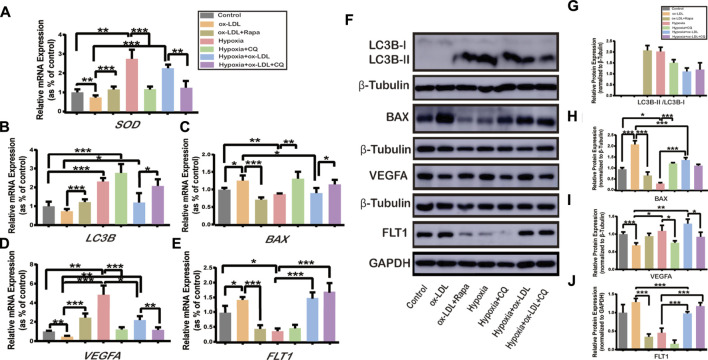
Relative expression of SOD, LC3B, BAX, VEGFA, and FLT1 in HTR8/SVneo cells. The relative mRNA expression of **(A)** SOD, **(B)** LC3B, **(C)** BAX, **(D)** VEGFA, and **(E)** FLT1 in HTR8/SVneo cells (*N* = 6). **(F)** Relative protein expression of LC3B, BAX, VEGFA, and FLT1 in HTR8/SVneo cells (*N* = 6). **(G)** Quantification of LC3B-II/LC3B-I, **(H)** BAX, **(I)** VEGFA, and **(J)** FLT1 protein expression normalized to β-Tubulin or GAPDH protein expression in HTR8/SVneo cells (*N* = 6). Data were expressed as normalized ratios (Control = 1). Set average Control = 1, and the other data were compared with it. The results were expressed as means ± standard errors. *p < 0.05, **p < 0.01, ***p < 0.001.

When the HTR8/SVneo cells were exposed to ox-LDL combined with Rapamycin (the ox-LDL + Rapa group), the expression of LC3B (Figure 4B,F,G) and VEGFA (Figure 4D,F,I) significantly increased compared with that in the ox-LDL group, and the expression of BAX (Figure 4C,F,H) and FLT1 (Figure 4E,F,J) decreased. The expressions of LC3B (Figure 4B,F,G) and VEGFA (Figure 4D,F,I) were increased, and those of BAX (Figure 4C,F,H) and FLT1(Figure 4E–J) were reduced when the cells were exposed to hypoxia (the Hypoxia and Hypoxia + ox-LDL groups) compared with that in the ox-LDL group. Chloroquine is the most effective compound for demonstrating the accumulation of LC3B-II, not representing autophagy activation (Brownfoot et al., 2016). Thus, when the cells were exposed to hypoxia combined with chloroquine, there was no difference in LC3B expression among the three groups (Figure 4B,F,G, the Hypoxia, Hypoxia + CQ, and Hypoxia + ox-LDL + CQ groups). The expression of BAX was increased (Figures 4C,F,H) and the expression of VEGFA was decreased (Figure 4D,F,I) in the Hypoxia + CQ group compared with the Hypoxia group and the Hypoxia + ox-LDL group compared with the Hypoxia + ox-LDL + CQ group. However, there was no difference of FLT1 expression between the Hypoxia group and the Hypoxia + ox-LDL group and the Hypoxia + ox-LDL group and the Hypoxia + ox-LDL + CQ group (Figure 4E,F,J).

### Possible Mechanism

**FIGURE 5:**
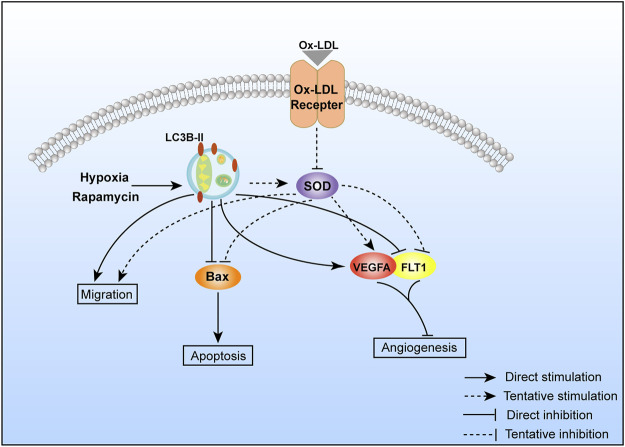
Possible molecular mechanism of autophagy regulating cell migration, apoptosis, and angiogenesis.

Autophagy activation could rescue ox-LDL–induced PE-like symptoms (migration declined, apoptosis, and angiogenesis impaired). The possible mechanism is shown in Figure 5.

## Discussion

EOPE is well known as a placental origin disease, which is characterized by placental shallow implantation and insufficient spiral artery recasting (von Dadelszen et al., 2003). Numerous theories have been proposed to explain the occurrence of preeclampsia, and it is now widely accepted that impaired angiogenesis and trophoblast apoptosis at the maternal–fetal interface play roles (Kasture et al., 2018; Raguema et al., 2020). In this study, we found that autophagy is repressed, trophoblast apoptosis is enhanced (Bax was upregulated), and angiogenesis is impaired (VEGFA was downregulated, and FLT1 was upregulated) in preeclampsia. We designed a series of experiments to show that autophagy activated by hypoxia could protect against the ox-LDL–mediated preeclampsia-like phenotype in HTR8/SVneo cells: migration declined, apoptosis (Bax was upregulated), and impaired angiogenesis (VEGFA was downregulated and FLT1 upregulated).

Autophagy is a process of lysosomal catabolism by eliminating dysfunctional proteins and providing alternative energy under stress. We confirmed that autophagy-related genes (LC3B and Beclin1) and VEGFA were downregulated, while Bax and FLT1 were upregulated in placentas from preeclamptic women (Figure 1), indicating that the autophagy pathway might play a significant role in preeclampsia. Rapamycin (autophagy activator) and chloroquine (autophagy inhibitor), which are autophagy-modifying agents, can alter the autophagy flux in cells (Nalbandian et al., 2015; Klionsky et al., 2016). To better understand the pathogenesis of hypoxia-induced autopahgy in preeclampsia, rapamycin and chloroquine were used to activate or inhibit the autophagy state. Related research demonstrated that hypoxia could induce HIF1α over-expression, thereby activating autophagy *via* the PI3K pathway (Yamanaka-Tatematsu et al., 2013).

When autophagy was activated, microtubule-associated protein 1A/1B-light chain 3 (LC3) was synthesized as proLC3 and the autophagy-related protein 4 (Atg4) protease processed proLC3 into LC3-I (Mizushima, 2007b). Upon the induction of autophagy, the exposed glycine residue of LC3-I conjugated to the highly lipophilic phosphatidylethanolamine moiety with Atg5 and Atg12 to generate LC3-II (Alirezaei et al., 2015). There were three types of LC3 sequences: LC3A, LC3B, and LC3C (He et al., 2003). The LC3B-II and LC3B-I ratios are sometimes recognized as markers of autophagy (Klionsky et al., 2016). Our data present LC3B mRNA expression, and the ratio of LC3B-II and LC3B-I was upregulated when treated with hypoxia and rapamycin, indicating that autophagy was activated (Figure 2C and Figure 4B,F,G).

In order to further verify our conjecture, ox-LDL was used to simulate the cellular microenvironment associated with preeclampsia in HTR8/SVneo cells. As shown in our data, ox-LDL–treated HTR8/SVneo cells exhibited preeclampsia-like phenotypic changes: declined migration (Figure 3C), impaired angiogenesis (VEGFA was downregulated and FLT1 upregulated), and apoptosis (Bax was upregulated) (Figure 3A), which were in accordance with the expression in the PE placenta. SOD expression was examined to verify ox-LDL stimulation–induced oxidative stress, which was reduced (Figure 2B). Our data showed that the treatment of ox-LDL exerts an inhibitory effect on VEGFA expression, which might lead to the inhibition of trophoblastic migration and angiogenesis. During pregnancy, VEGFA upregulation could enhance the migration of trophoblastic cells, contributing to angiogenesis (Andraweera et al., 2012). Some other studies showed that VEGFA expression from the placenta is reduced in preeclamptic women (Jarvenpaa et al., 2007; Andraweera et al., 2012). FLT1 is a potent antiangiogenic protein with a high affinity for VEGFA and could block angiogenesis and could be produced by the placenta, presenting in villous and extravillous trophoblasts (Austdal et al., 2019). Some researchers believed that the increased FLT1 expression in the preeclamptic placenta was mediated by HIF-1 (hypoxia-inducible factor 1) (Nevo et al., 2006).

We observed that VEGFA upregulation (Figure 4D,F,I) and the migration ability were enhanced (Figures 3C,D) in the following: 1) trophoblasts treated with ox-LDL + Rapa, 2) trophoblasts treated with hypoxia, and 3) trophoblasts treated with hypoxia + ox-LDL compared with hypoxia + ox-LDL + CQ. The FLT1 expression was downregulated (Figure 4E–J) in the following: 1) trophoblasts treated with ox-LDL + Rapa, 2) trophoblasts treated with hypoxia, and 3) trophoblasts treated with hypoxia + CQ. All of the above results indicated that the ability of angiogenesis and enhanced cell migration ability are potentially related to autophagy activation. Some studies showed that the activation of autophagy could upregulate VEGFA expression (Dai et al., 2018; Liang et al., 2018; Fan et al., 2018). In our data, the VEGFA expression was downregulated when cells were exposed to chloroquine (Figure4D,F,I, the Hypoxia + CQ and Hypoxia + ox-LDL + CQ groups). On the whole, the autophagy pathway plays an important role in the regulation of VEGFA and FLT1 under hypoxic conditions. Liang et al. (2018) revealed that autophagy upregulated VEGFA expression in lung cancer cells *via* activation of the JAK2/STAT3 pathway (Liang et al., 2019). Related research also showed that the activation of autophagy then mediated VEGFA upregulation by activating the SRC/STAT3 pathway (Xu et al., 2016).

From our data, ox-LDL could increase BAX expression, indicating that ox-LDL might mediate the apoptosis progress in trophoblasts. Apoptosis is an important mechanism in preeclampsia, which is defined as a programmed cell death (Elmore, 2007). It was reported that trophoblast cell apoptosis increased in the preeclampsia placenta compared with the normal placenta (Allaire et al., 2000). Bax is a well-known proapoptotic member of the Bcl-2 family, which is an important factor in apoptotic pathways (Sharp et al., 2014). Apoptosis induced by ox-LDL could be found in neutral sphingomyelinase and arterial smooth muscle cells *via* the mitochondrial apoptotic pathway and the death-receptor (Fas/FasL) apoptotic pathway (Chatterjee et al., 2021). From our data, autophagy induced by hypoxia could reduce BAX expression. Chen, F. et al. have found that ATG16L1 knockdown inhibited autophagy flux and increased BAX protein levels by suppressing BAX degradation (Chen et al., 2020). Some studies demonstrated that autophagy-induced morphological feature changes might occur prior to apoptotic cell death, which could represent the early phase of apoptosis (Elmore, 2007). In our experiment, the early apoptosis ratio decreased when cells were exposed to ox-LDL and the advanced apoptosis ratio increased. When the cells experienced hypoxia, the early apoptosis ratio and the advanced apoptosis ratio were not different compared with those in the control group, which indicated that hypoxia-induced autophagy could reduce cell apoptosis. Thus, we assumed that cell apoptosis was potentially interrupted by autophagy activation. Related research showed that autophagy could resist apoptosis *via* the AMP-activated protein kinase/mammalian target of the rapamycin signaling pathway (Xia and Hou, 2016).

There were also some limitations in this study. First, in our hospital, premature deliveries (delivery time <34 weeks) mainly included spontaneous preterm delivery, preterm premature membrane rupture, or preterm birth for medical and obstetrical indications. Due to these conditions, early placenta tissues from normal pregnancies (<34 weeks) without influence from disease or drugs could not be obtained. Thus, the gestational age of normotensive pregnancy placentae matched that of PE pregnancy placentae. Second, the effect of autophagy on the regulation of VEGFA or other genes in EOPE could not be determined from this study and requires further research. This study investigated the fact that autophagy is repressed in preeclampsia, and autophagy activated by hypoxia could rescue the ox-LDL–mediated preeclampsia-like phenotype in HTR8/SVneo cells: declined migration, apoptosis (Bax was upregulated), and impaired angiogenesis (VEGFA was downregulated and FLT1 upregulated). Further exploration was needed to study the molecular mechanisms in autophagy and the effect of autophagy activation on apoptosis, angiogenesis, and migration activity in trophoblasts, in an attempt to provide some new ideas in preeclampsia prevention.

## Data Availability

The original contributions presented in the study are included in the article/Supplementary Material; further inquiries can be directed to the corresponding authors.
