# Supplementary figures and images for: Autophagy Activation by Hypoxia Regulates Angiogenesis and Apoptosis in Oxidized Low-Density Lipoprotein-Induced Preeclampsia
Source: Front Mol Biosci. 2021 Sep 9;8:709751. doi: 10.3389/fmolb.2021.709751 (PMC8458810; doi:10.3389/fmolb.2021.709751)

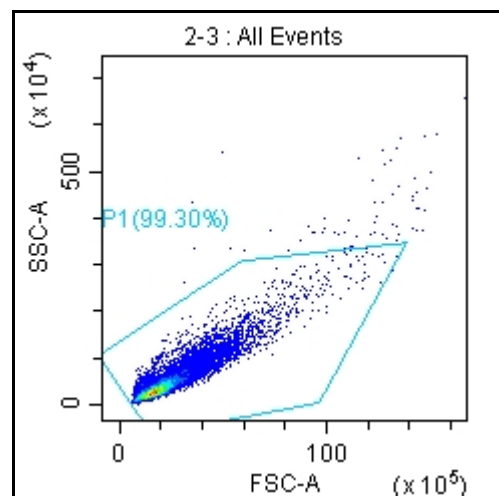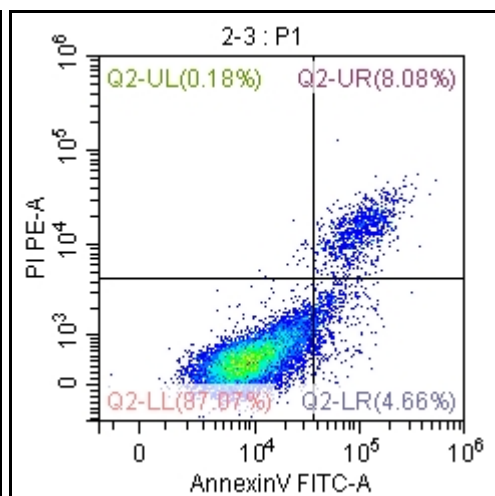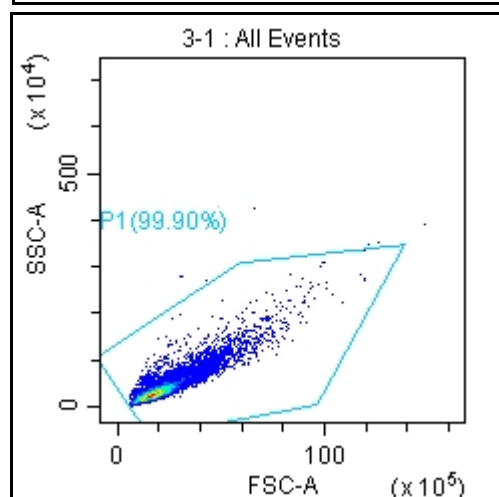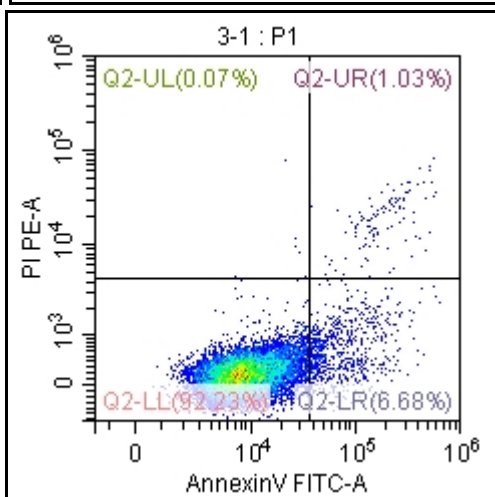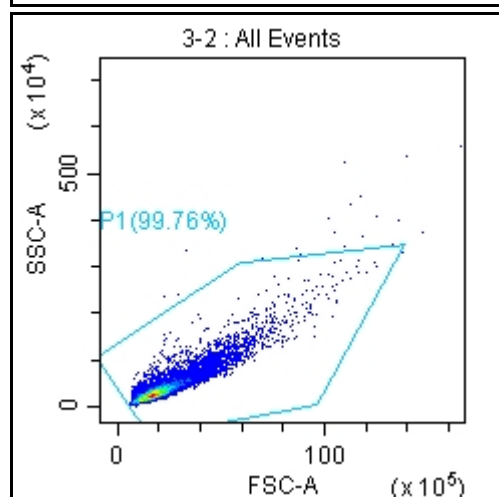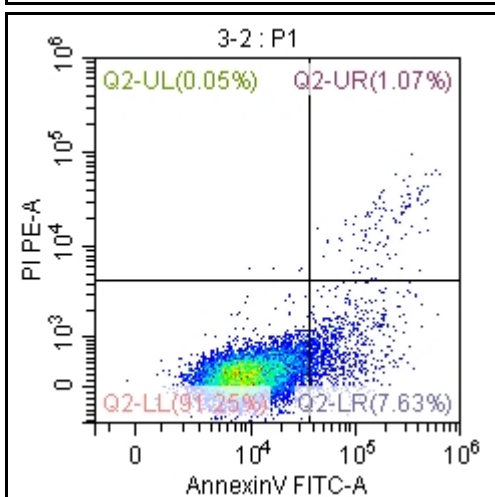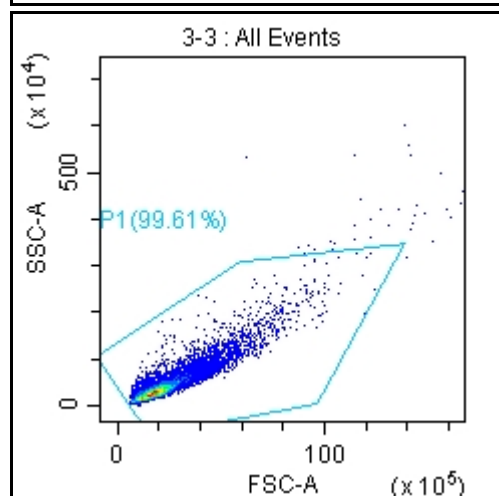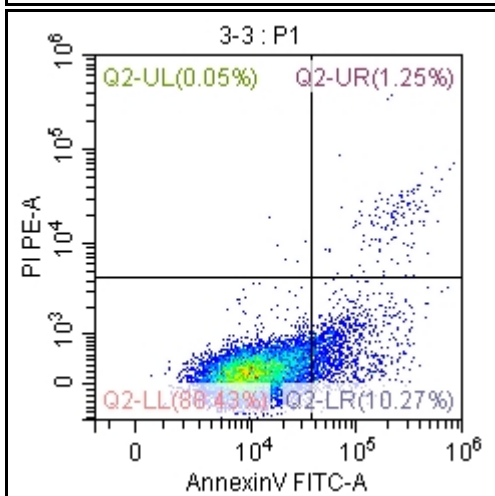

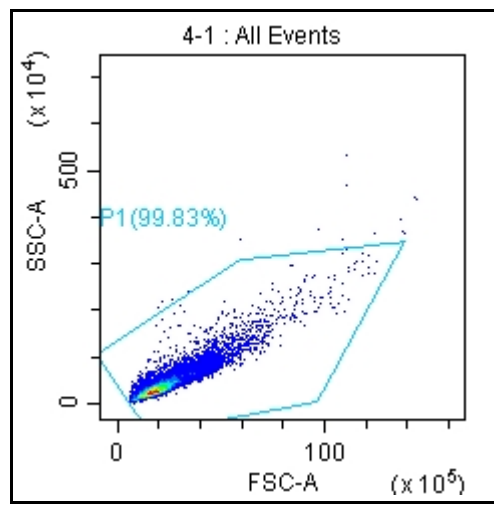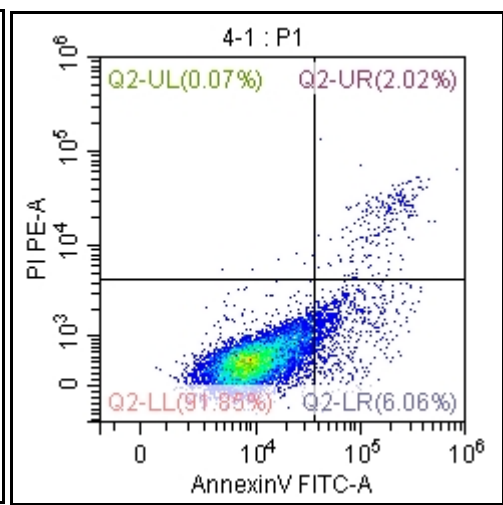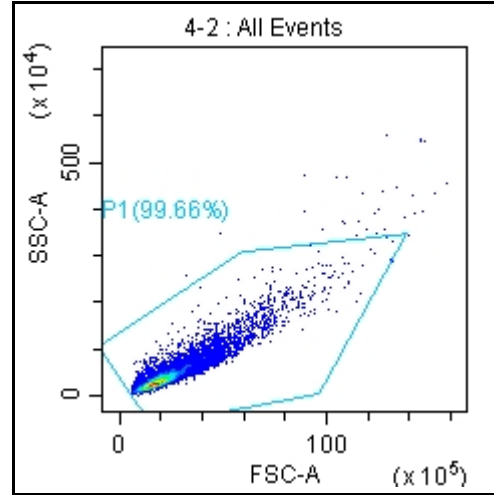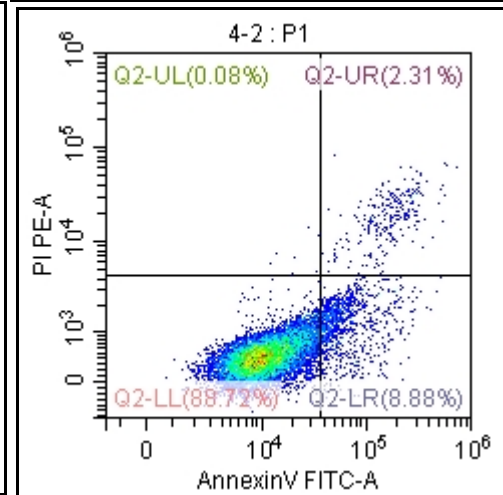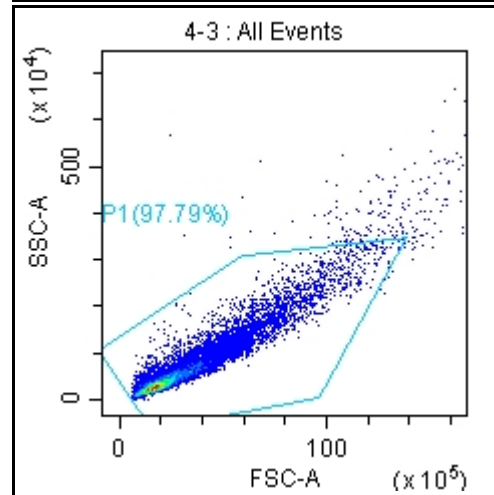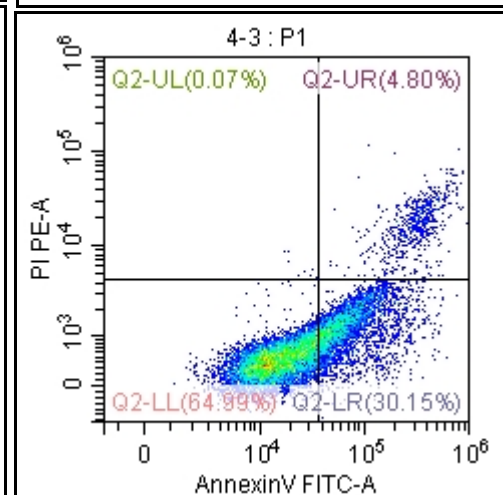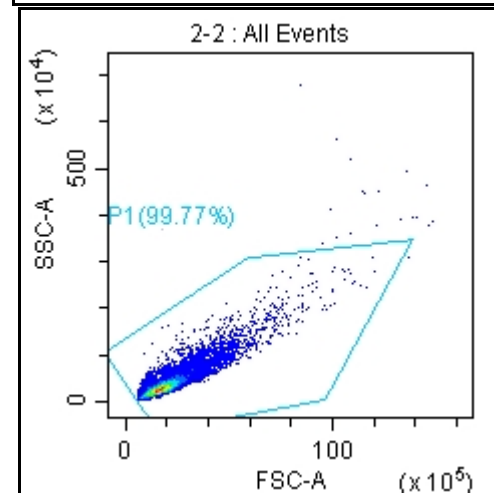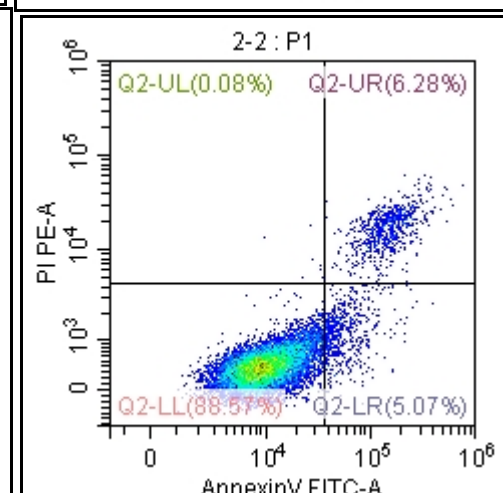

Supplement: Supplementary file 1 [file DataSheet2.PDF]

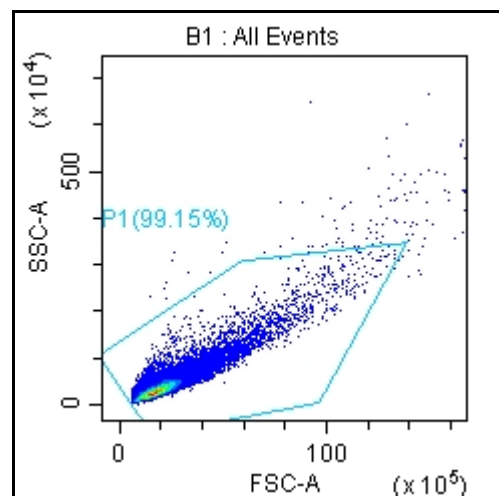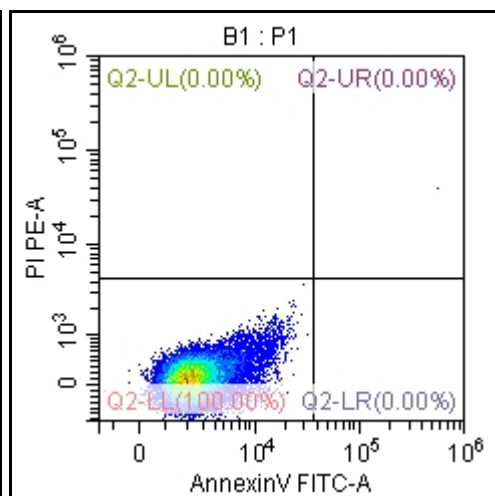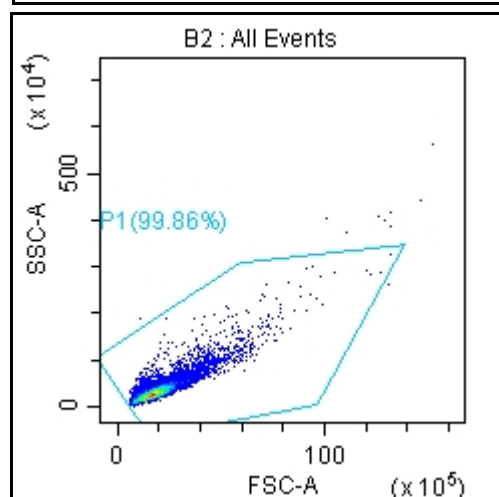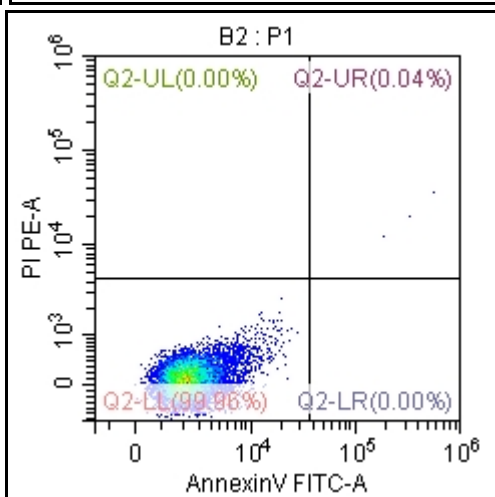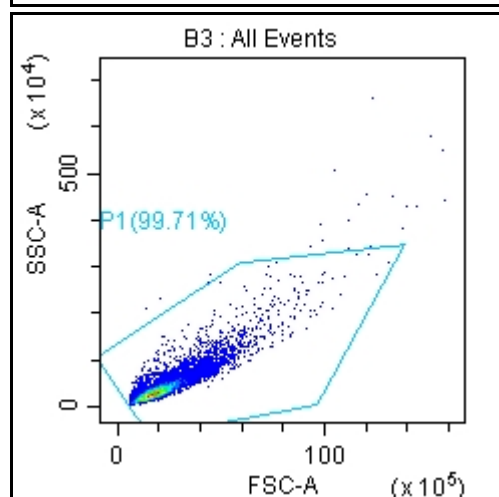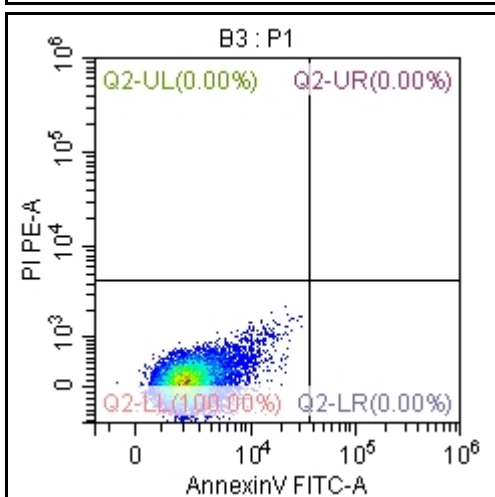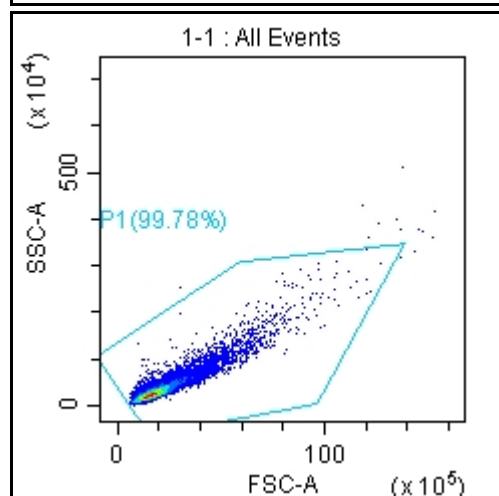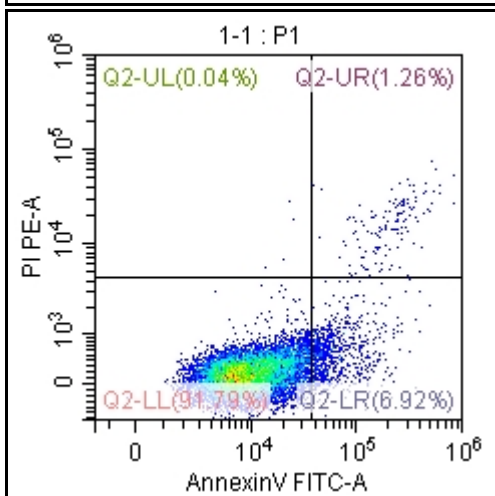

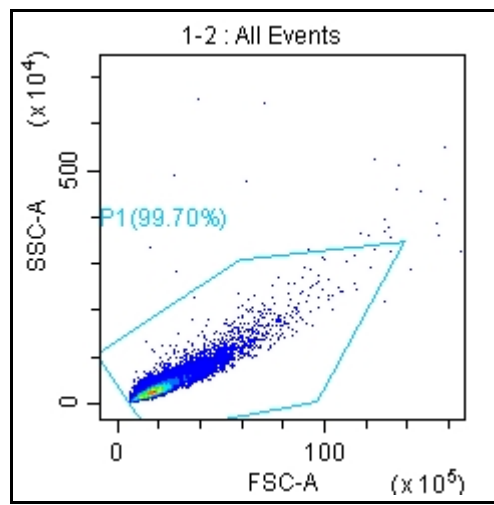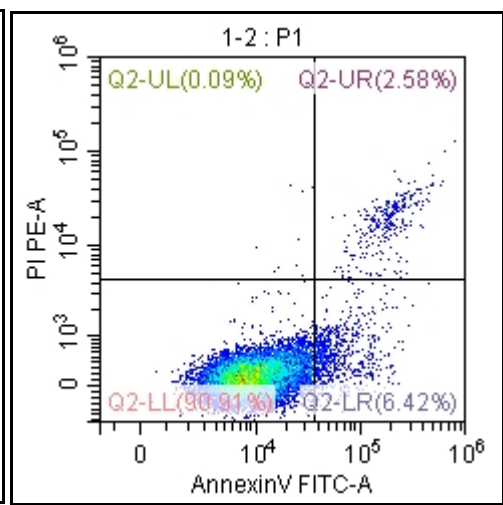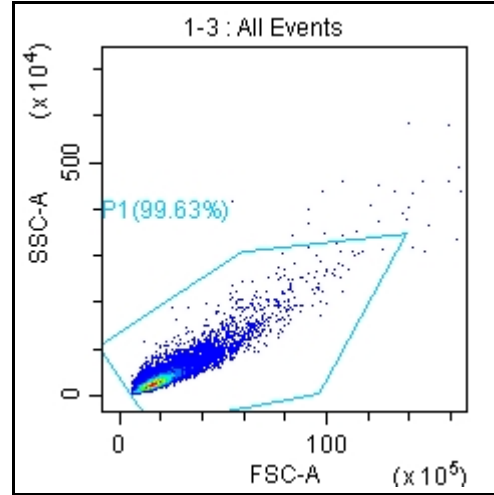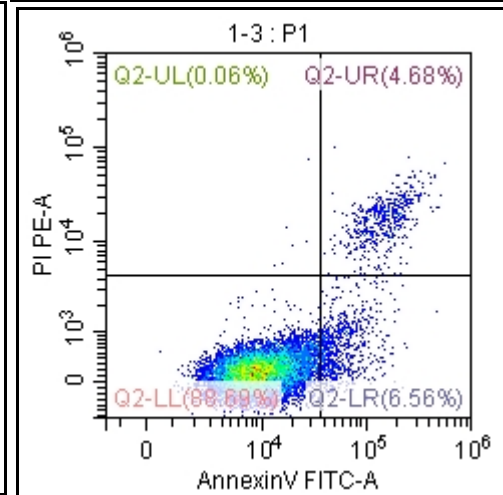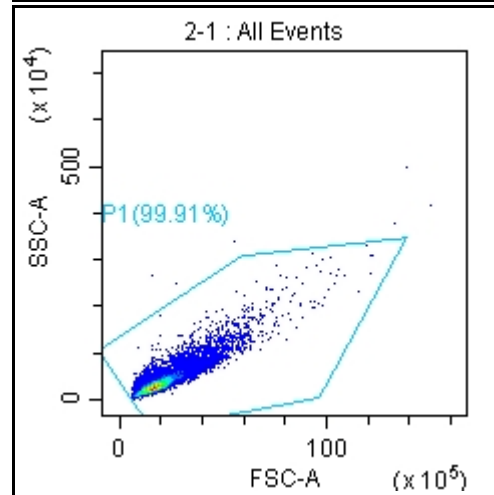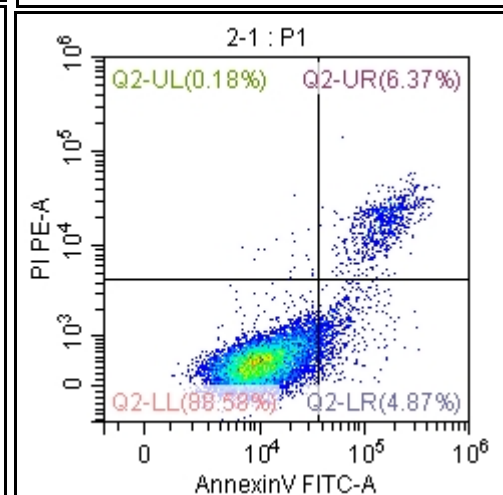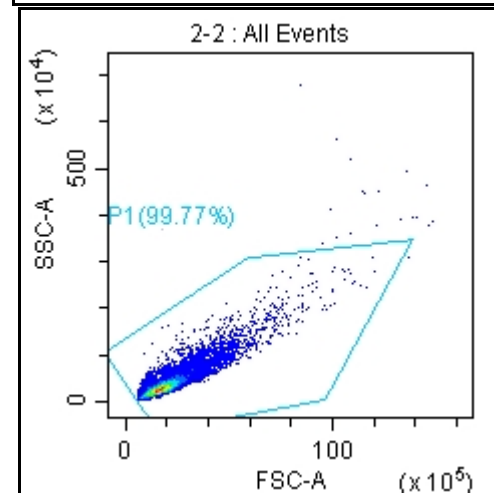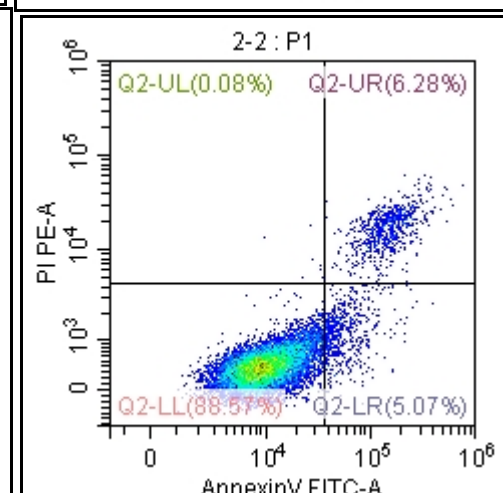

Supplement: Supplementary file 2 [file DataSheet3.PDF]
