# Supplementary material for: Autophagy Activation by Hypoxia Regulates Angiogenesis and Apoptosis in Oxidized Low-Density Lipoprotein-Induced Preeclampsia
Source: Front Mol Biosci. 2021 Sep 9;8:709751. doi: 10.3389/fmolb.2021.709751 (PMC8458810; doi:10.3389/fmolb.2021.709751)

Figure 1 A


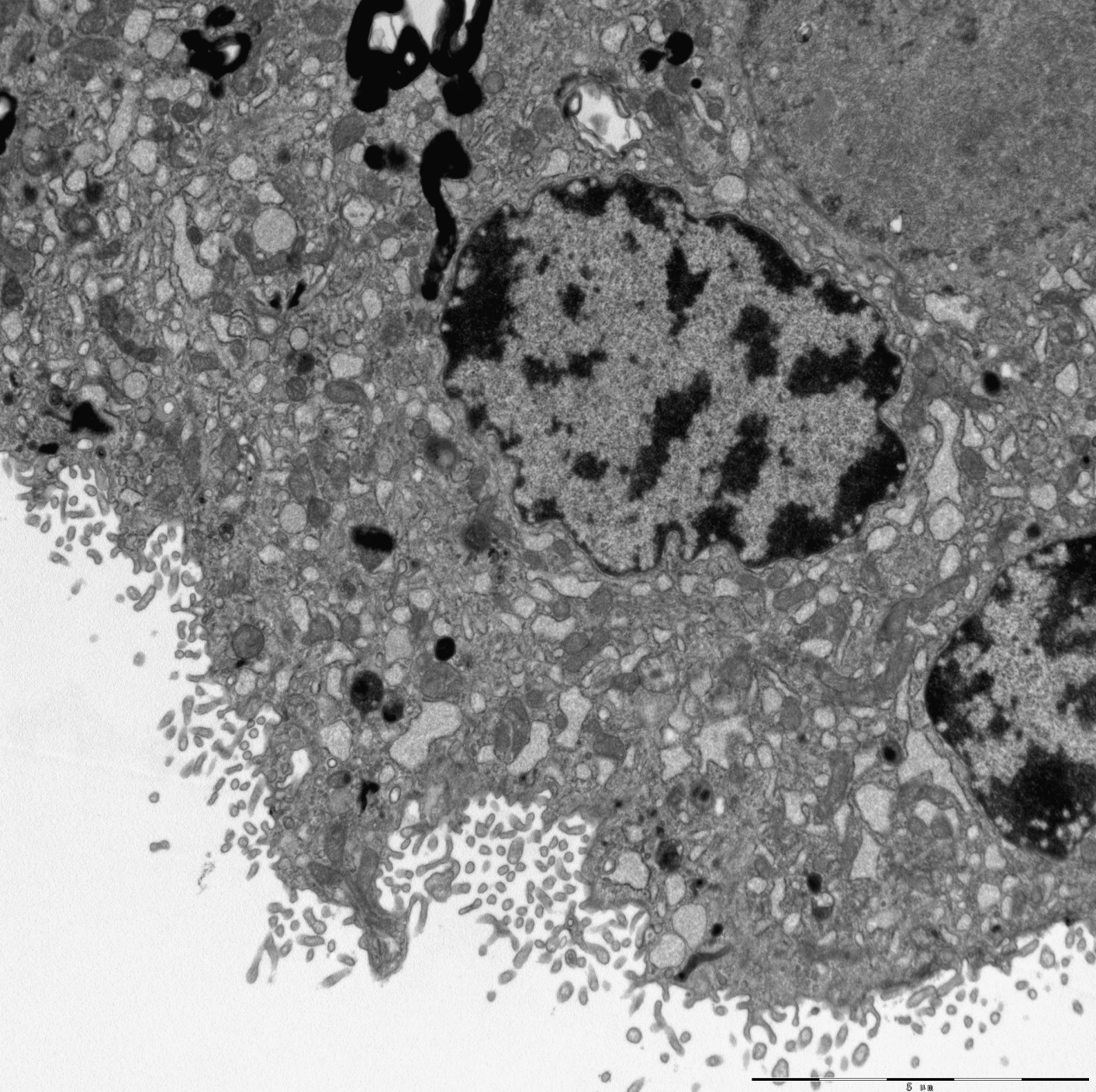


Figure1B
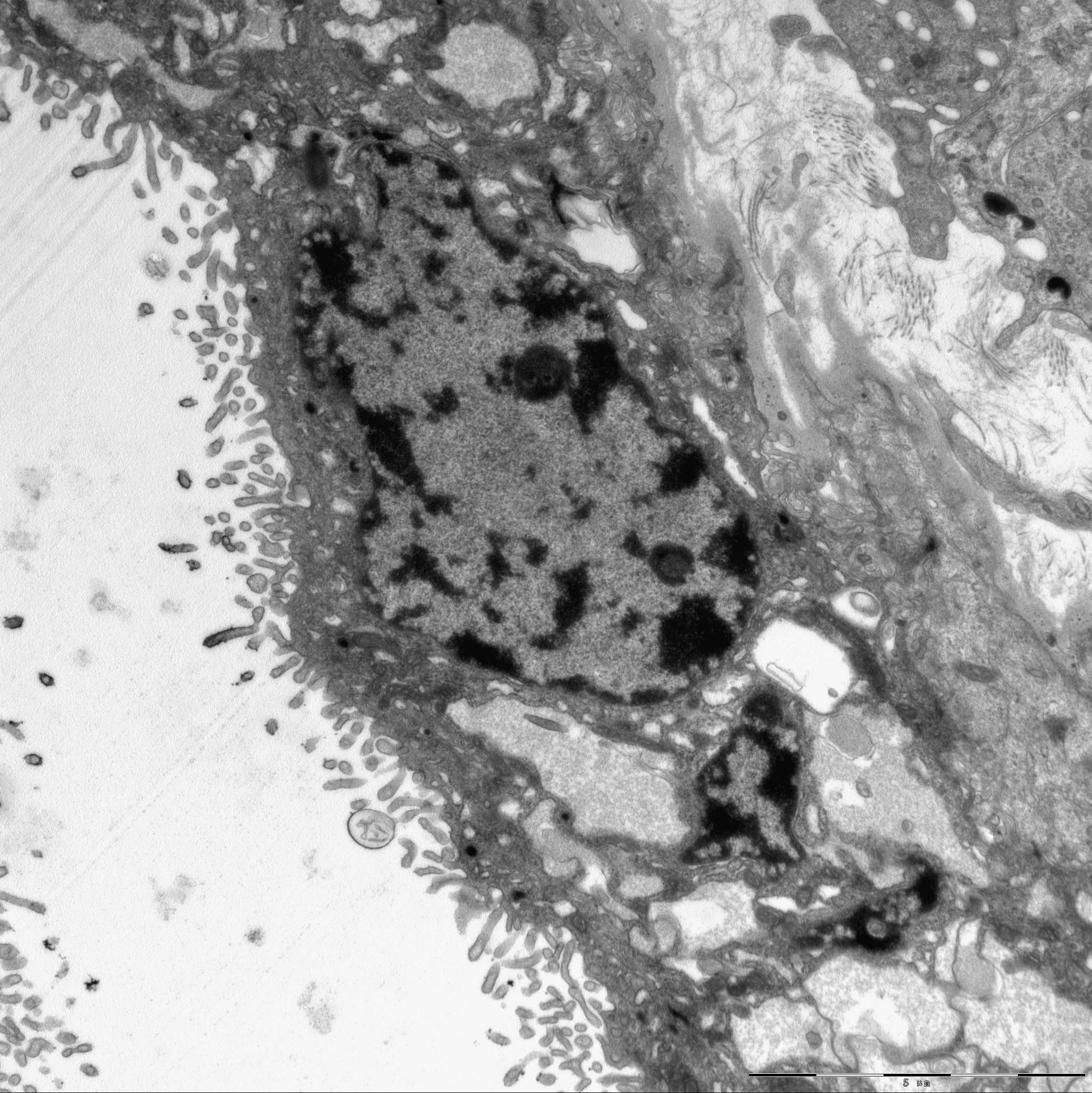


Figure1C
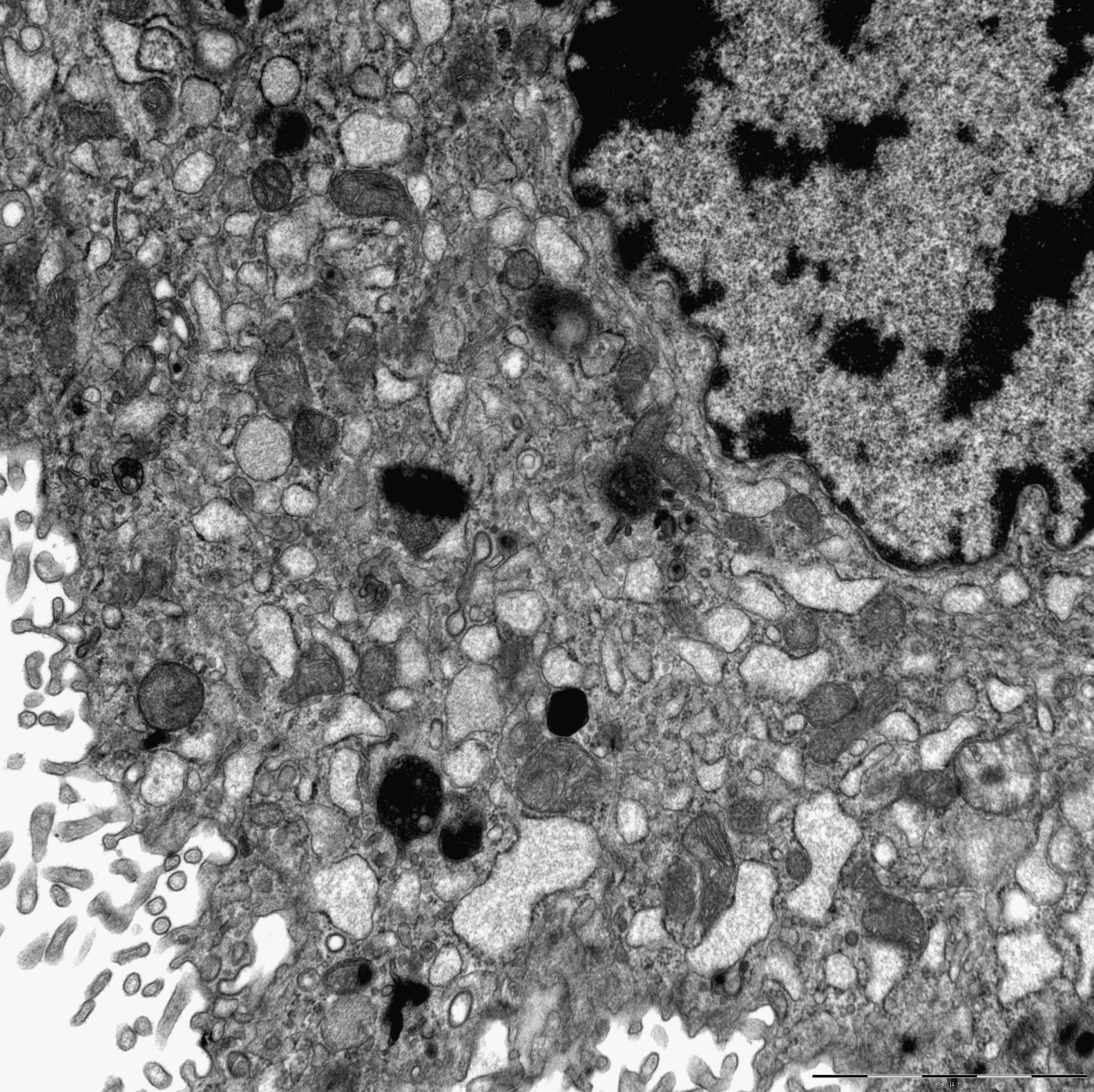


Figure1 D


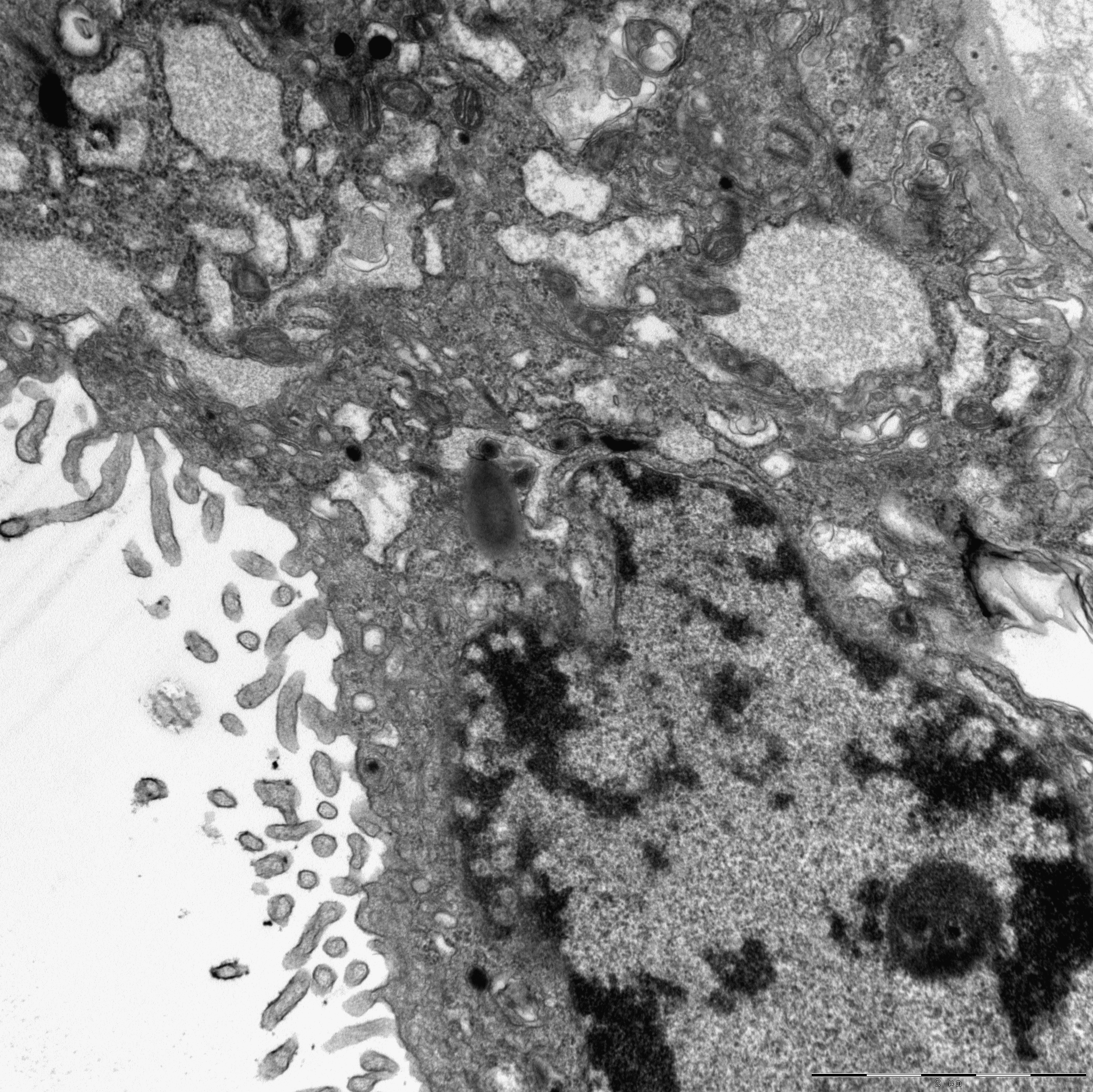


Figure1 H： LC3B+β-Tubulin


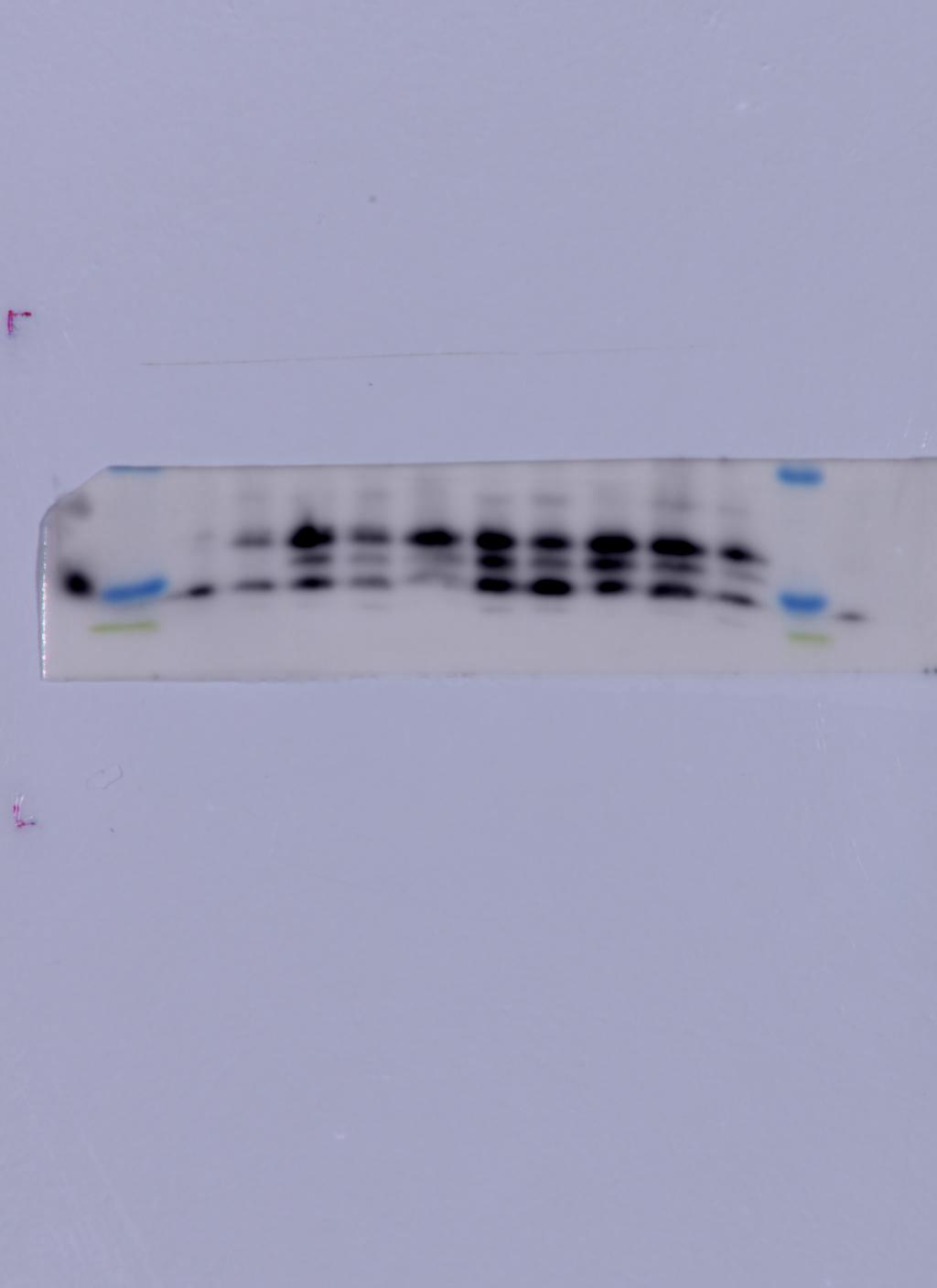


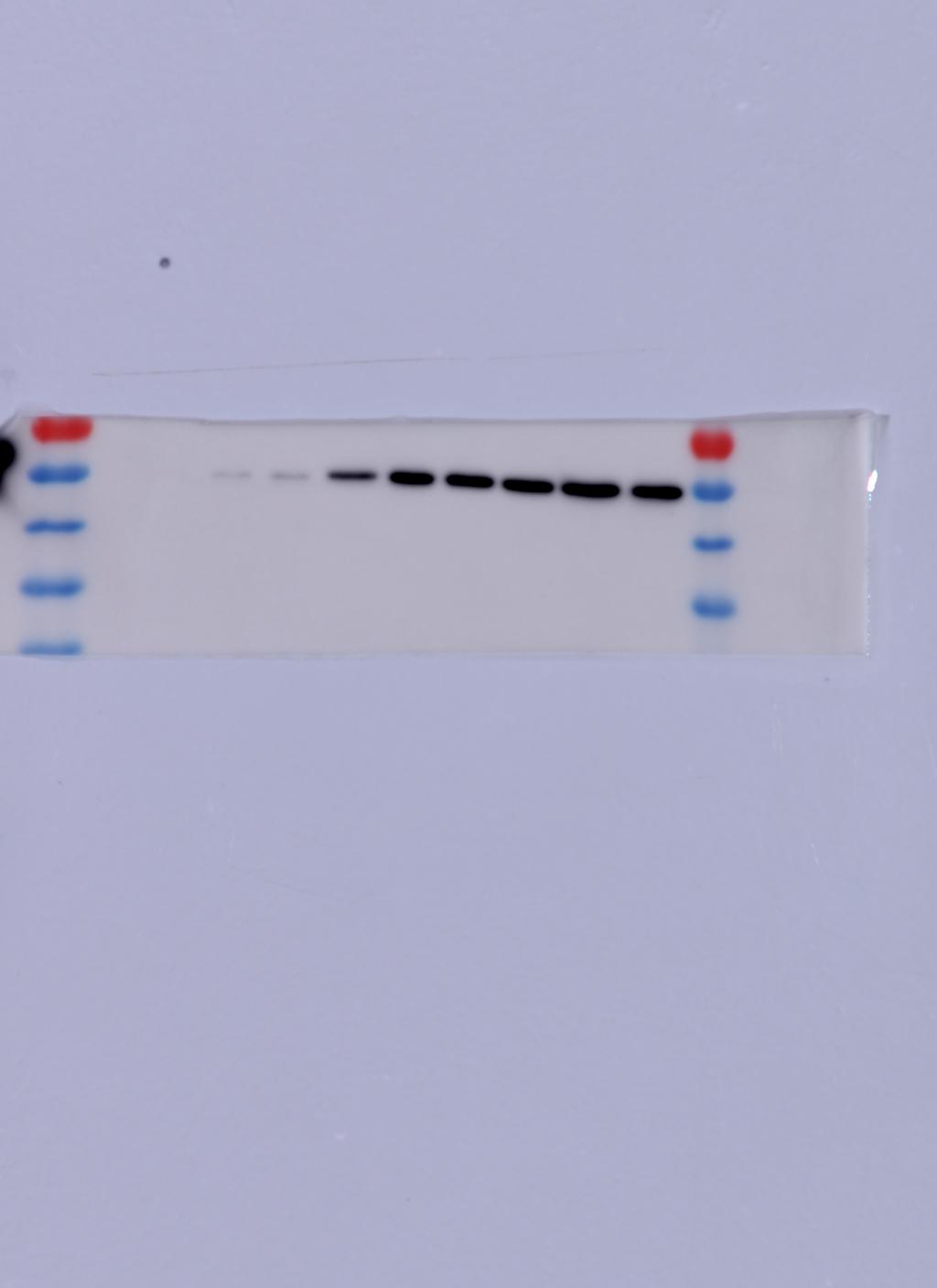


Figure1 H：Beclin1+Gapdh


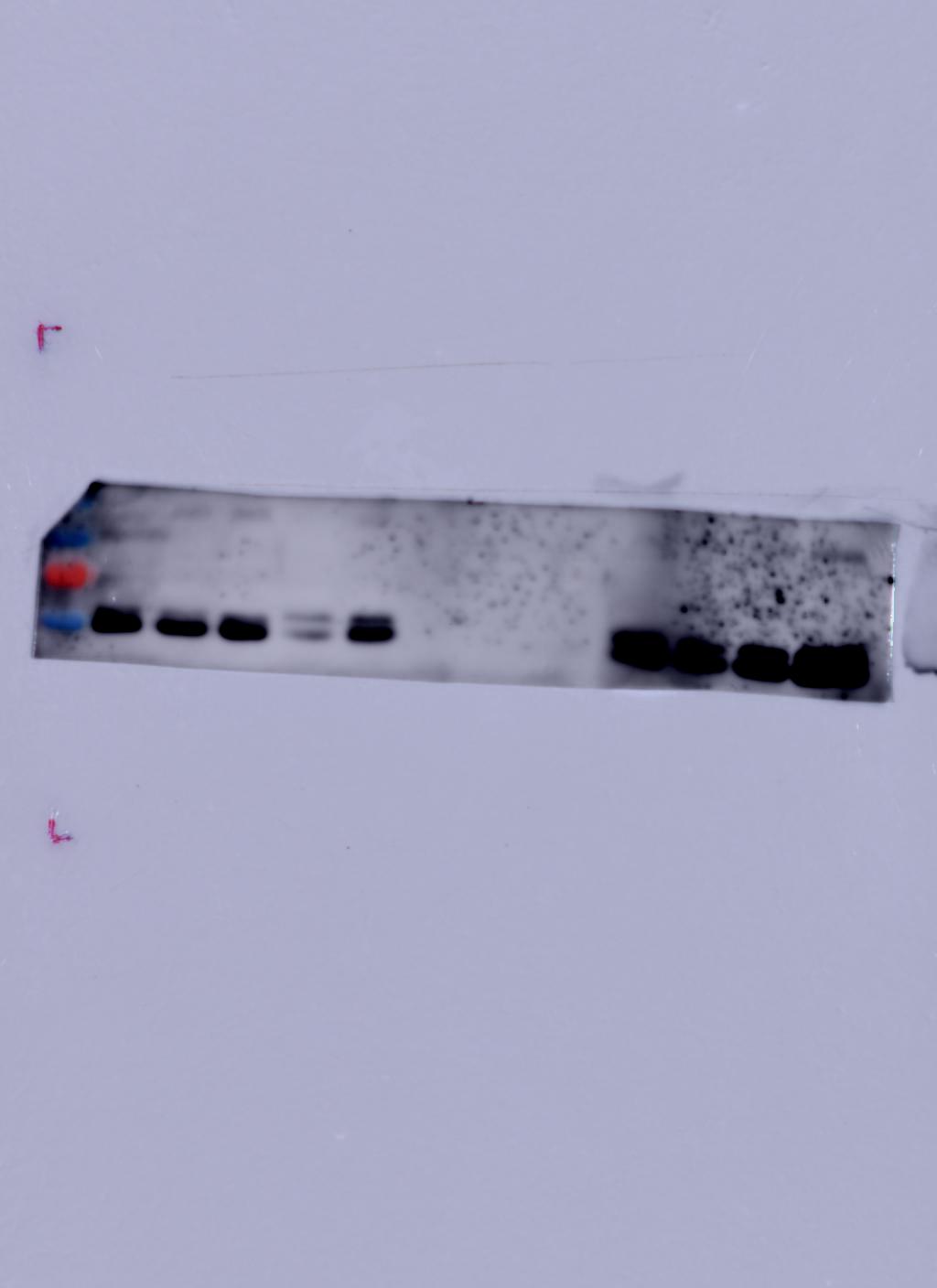


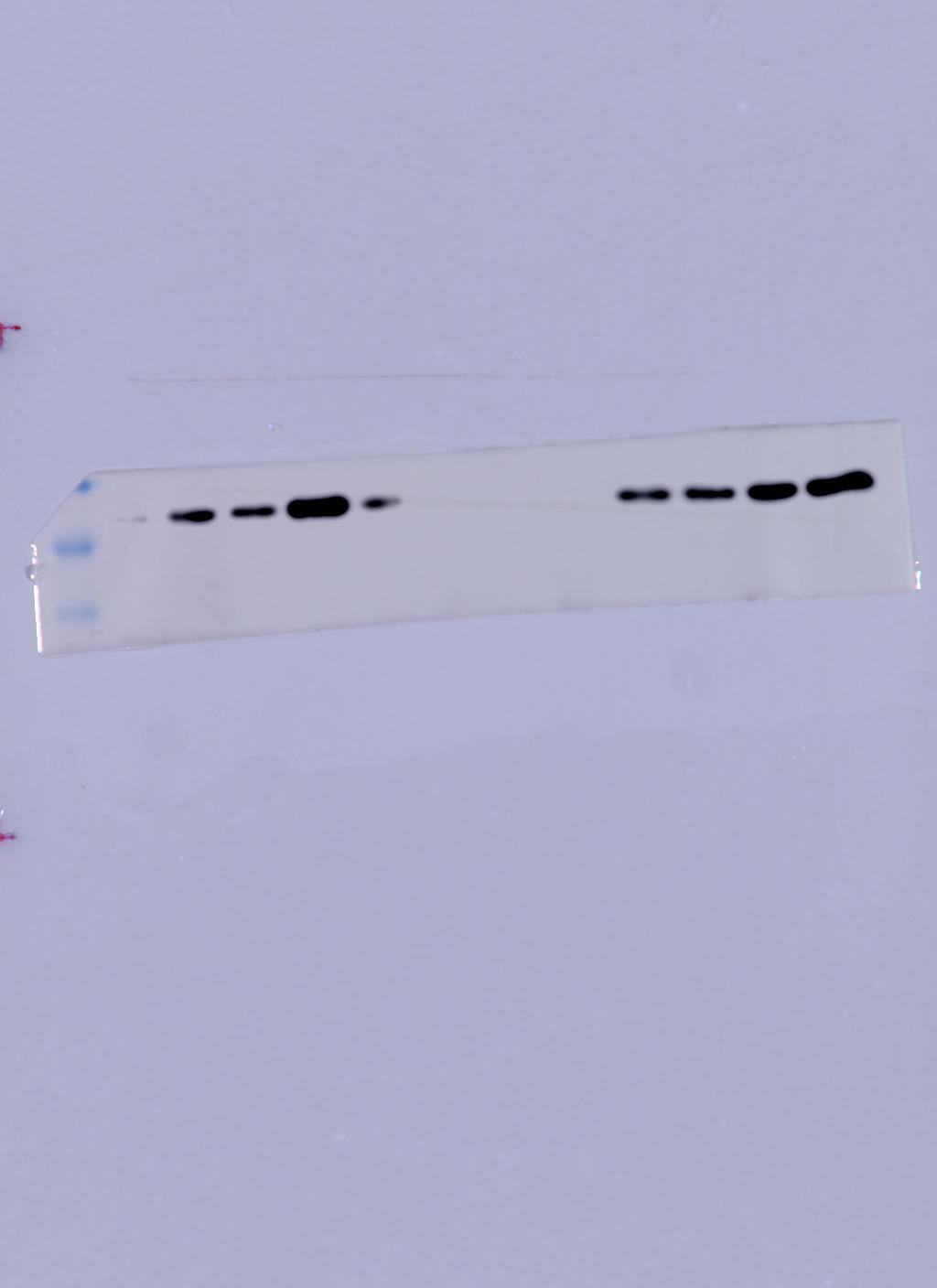


Figure1 H： Bax+Gapdh


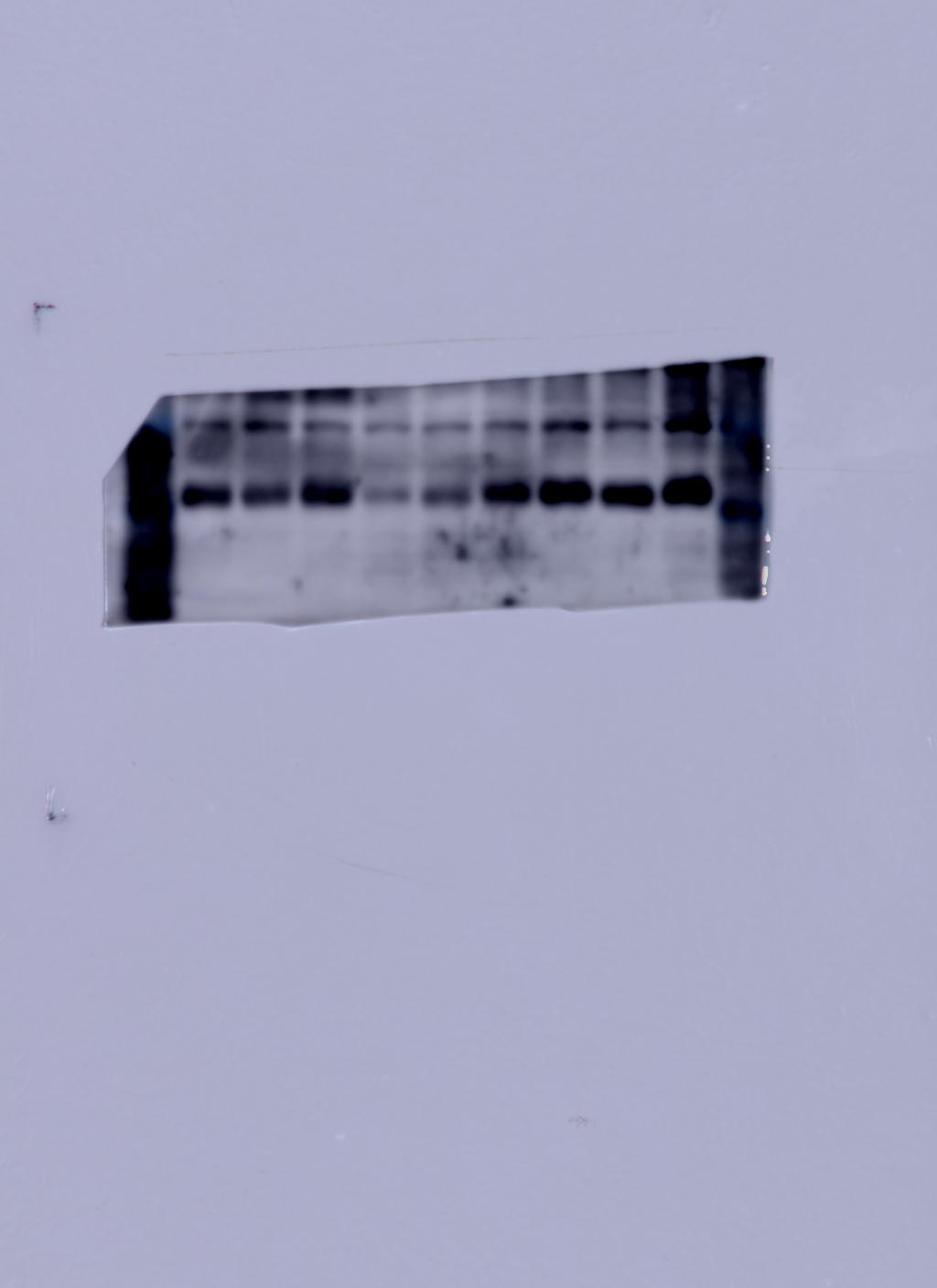


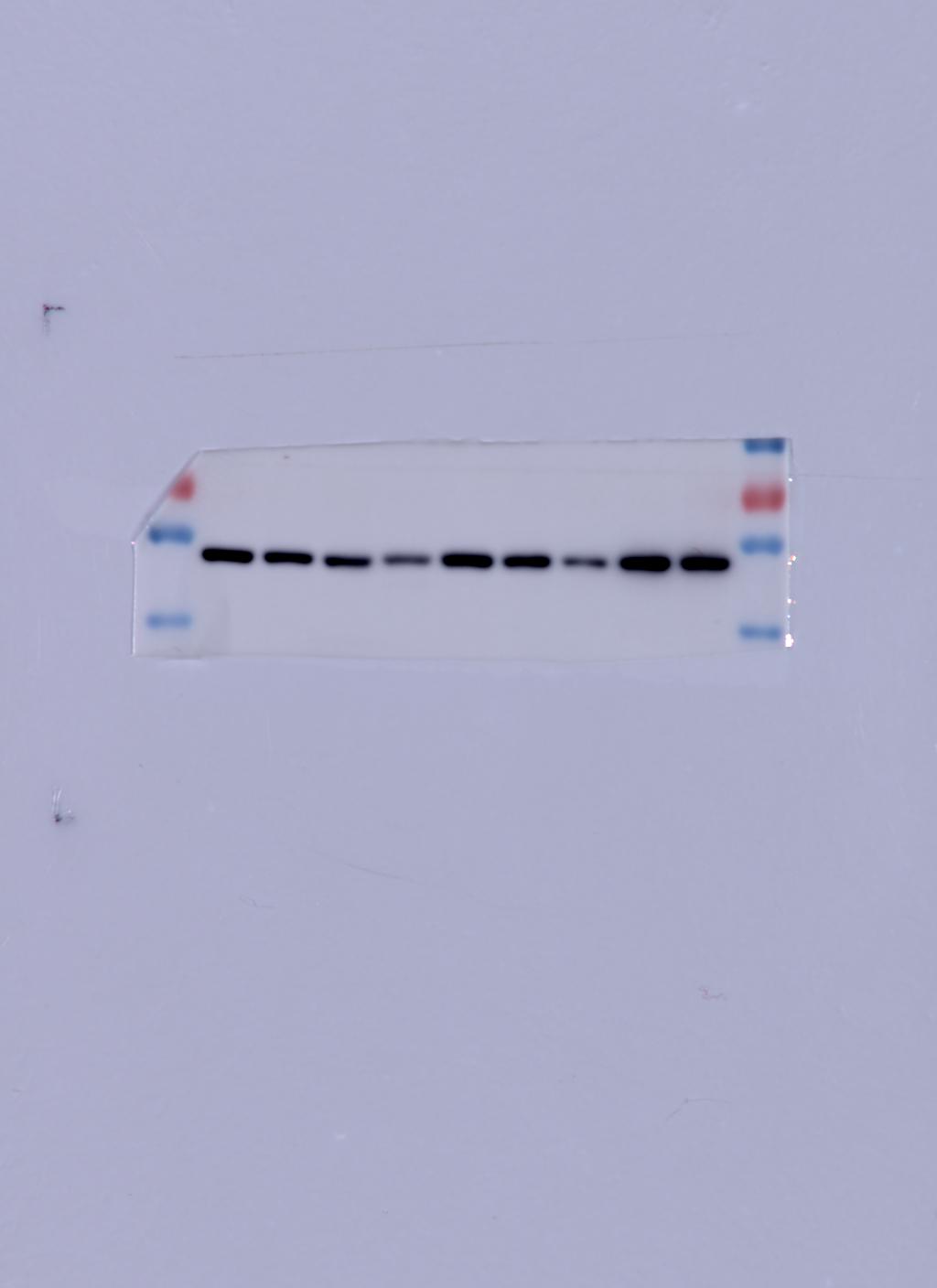


Figure1 H： VEGF+β-Tubulin


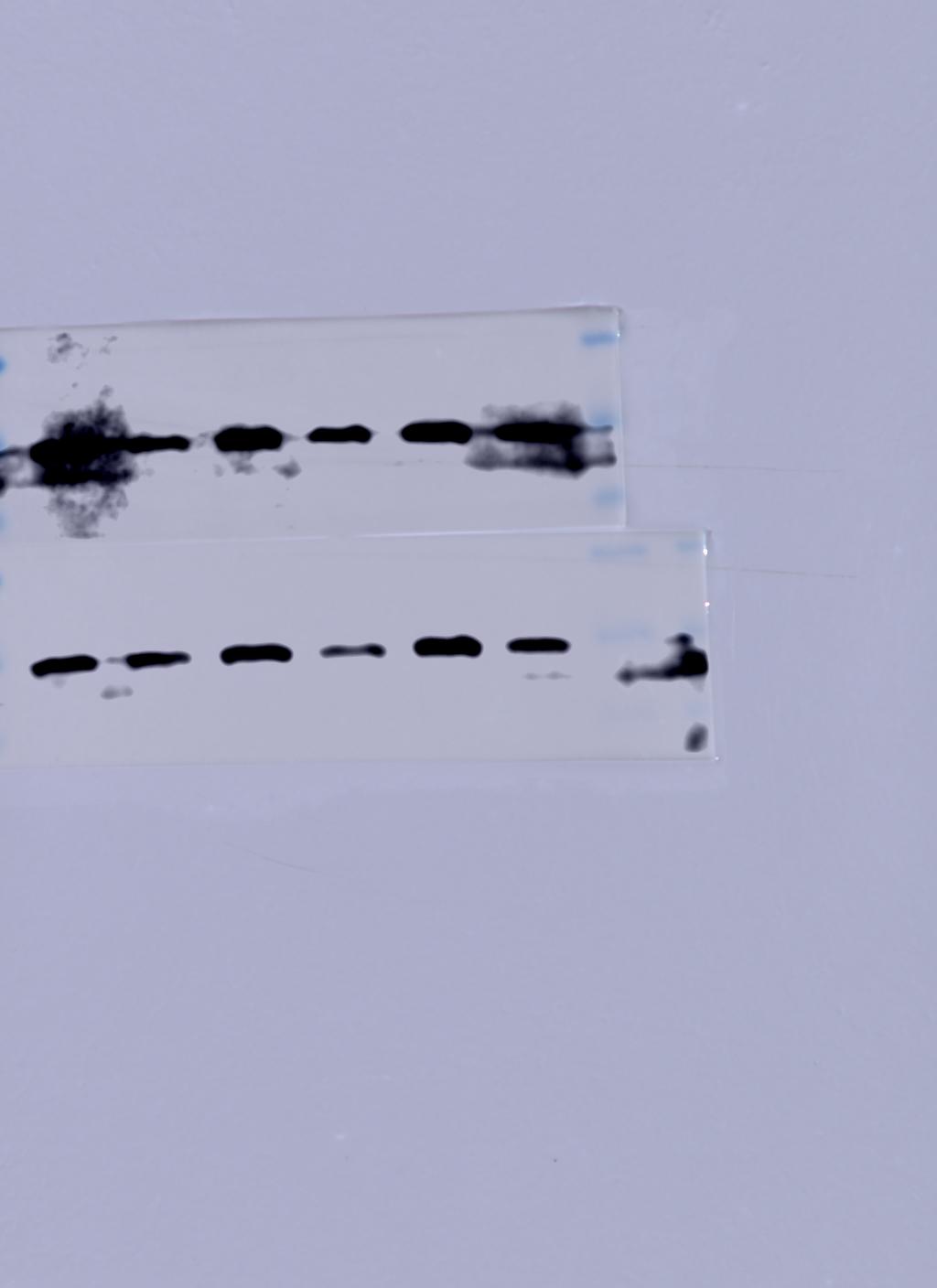


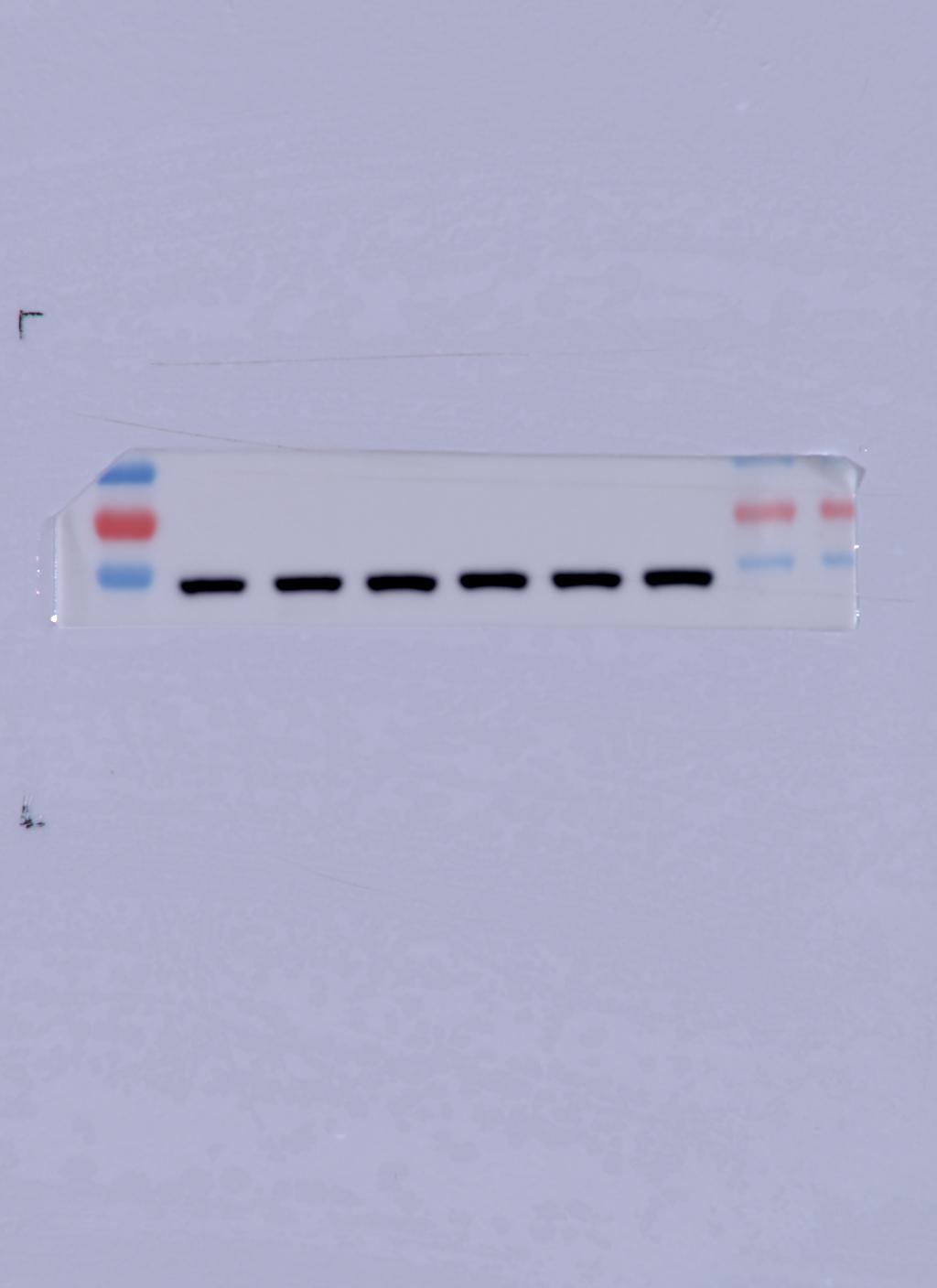


Figure1 H： FLT1+β-Tubulin


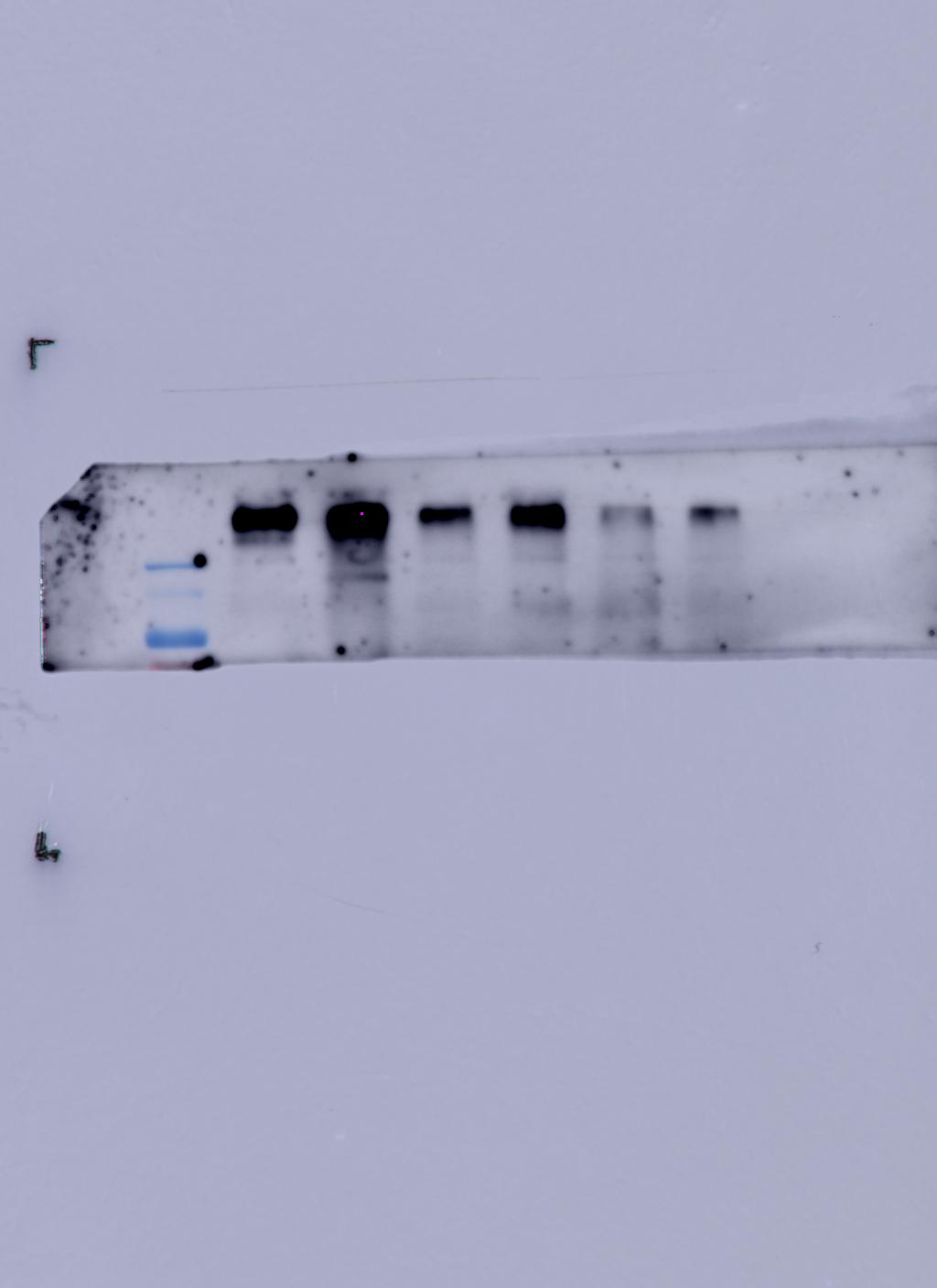


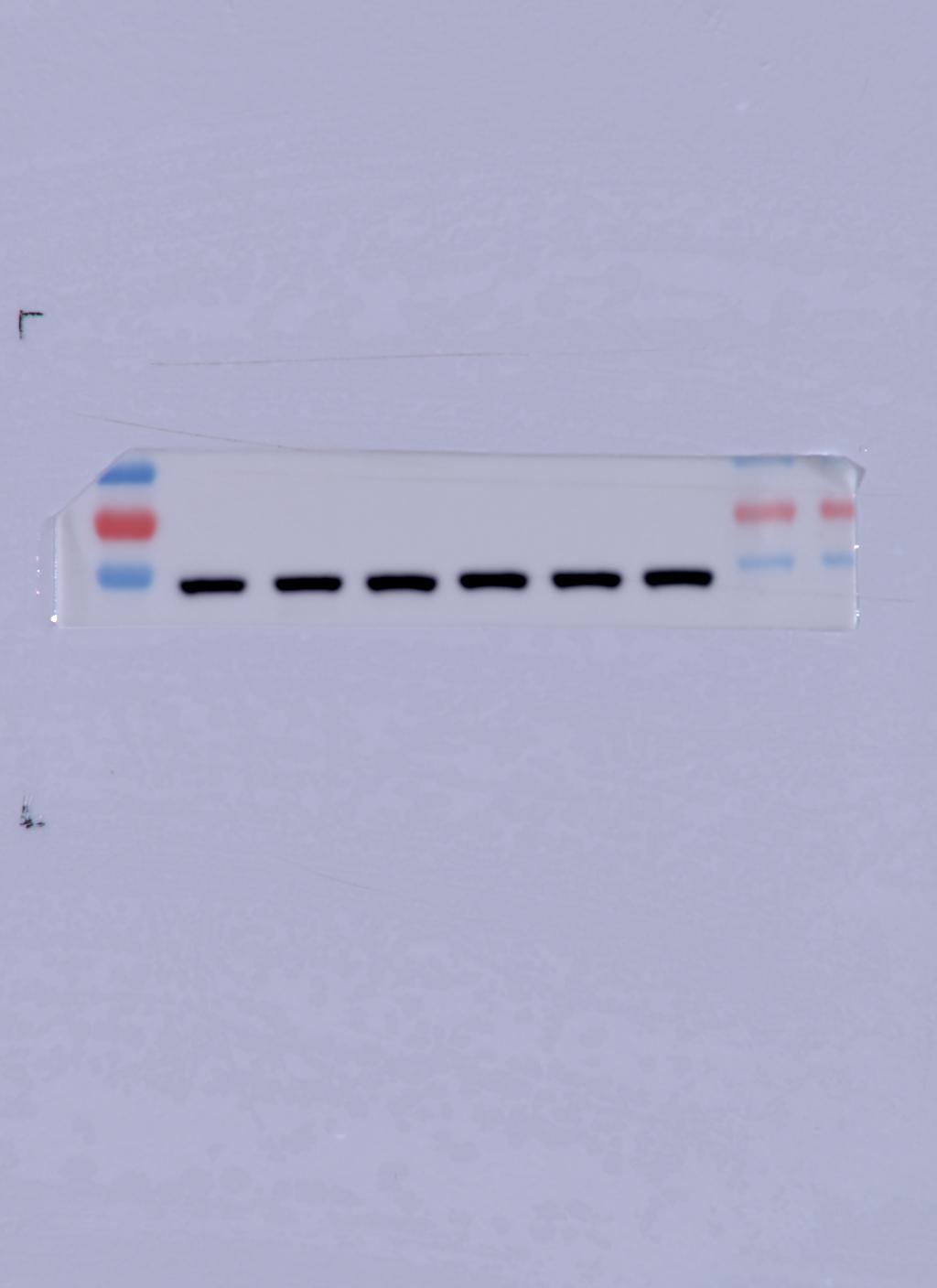


Figure2 C ：LC3+P62+β-actin


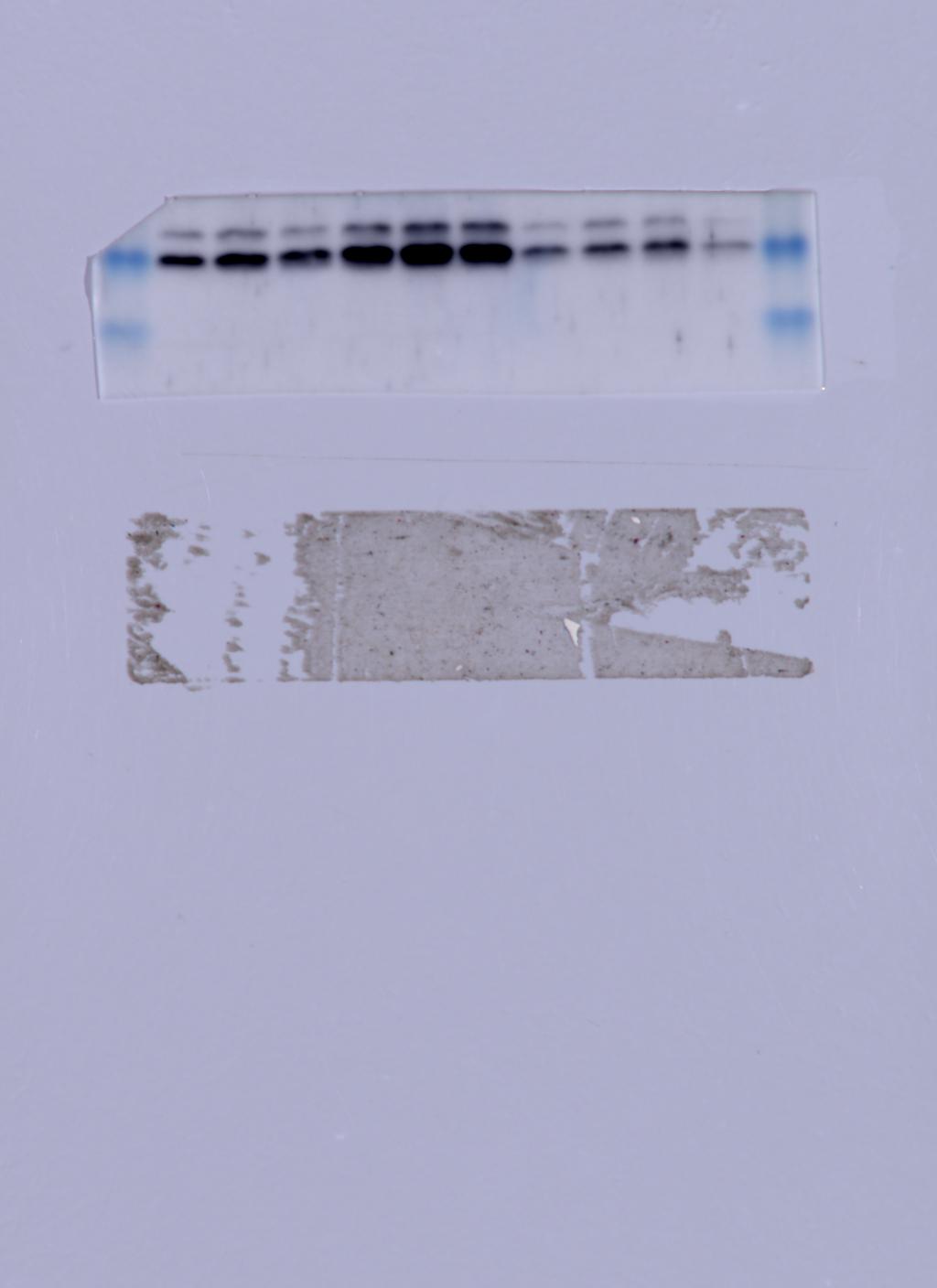


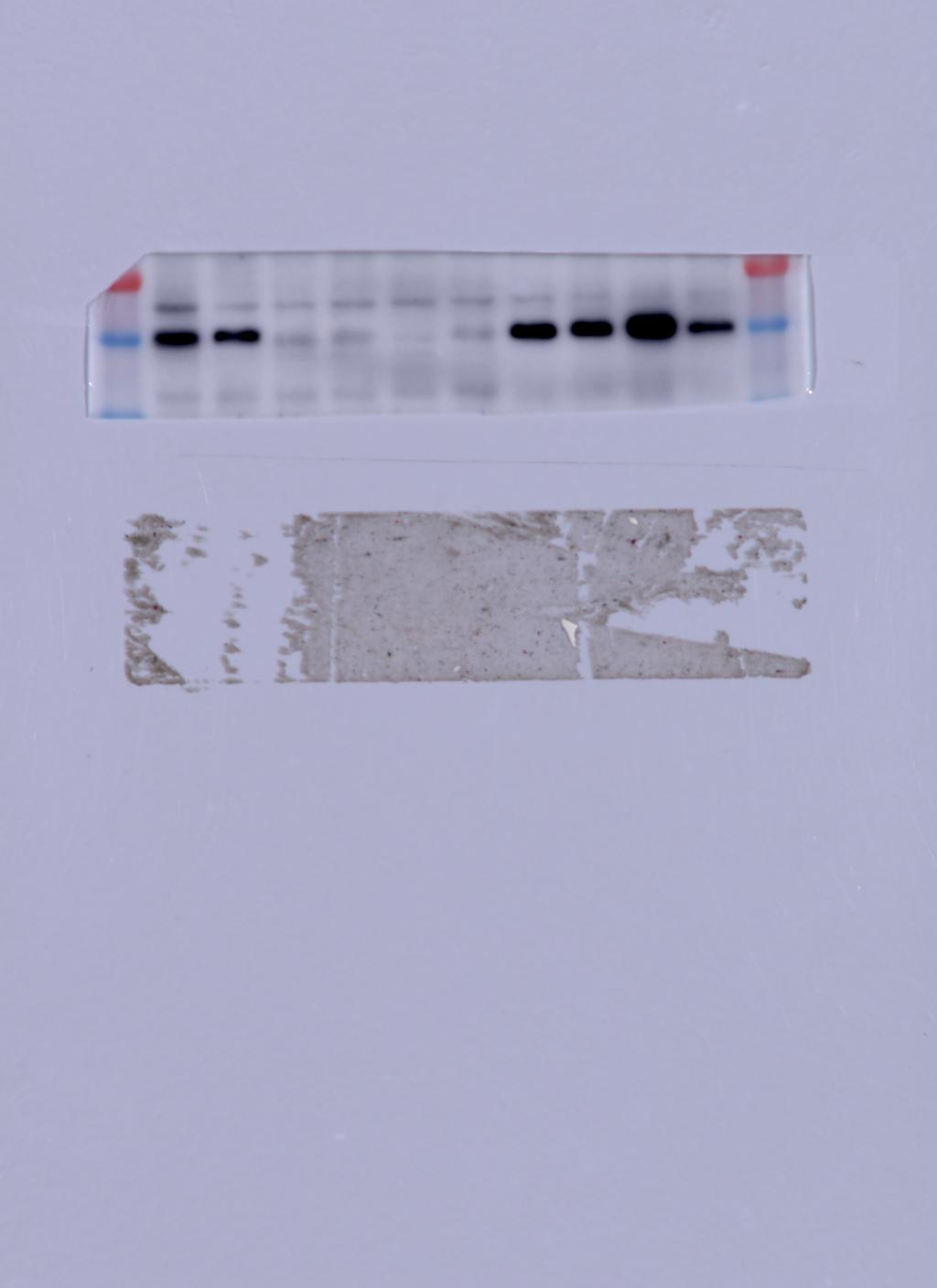


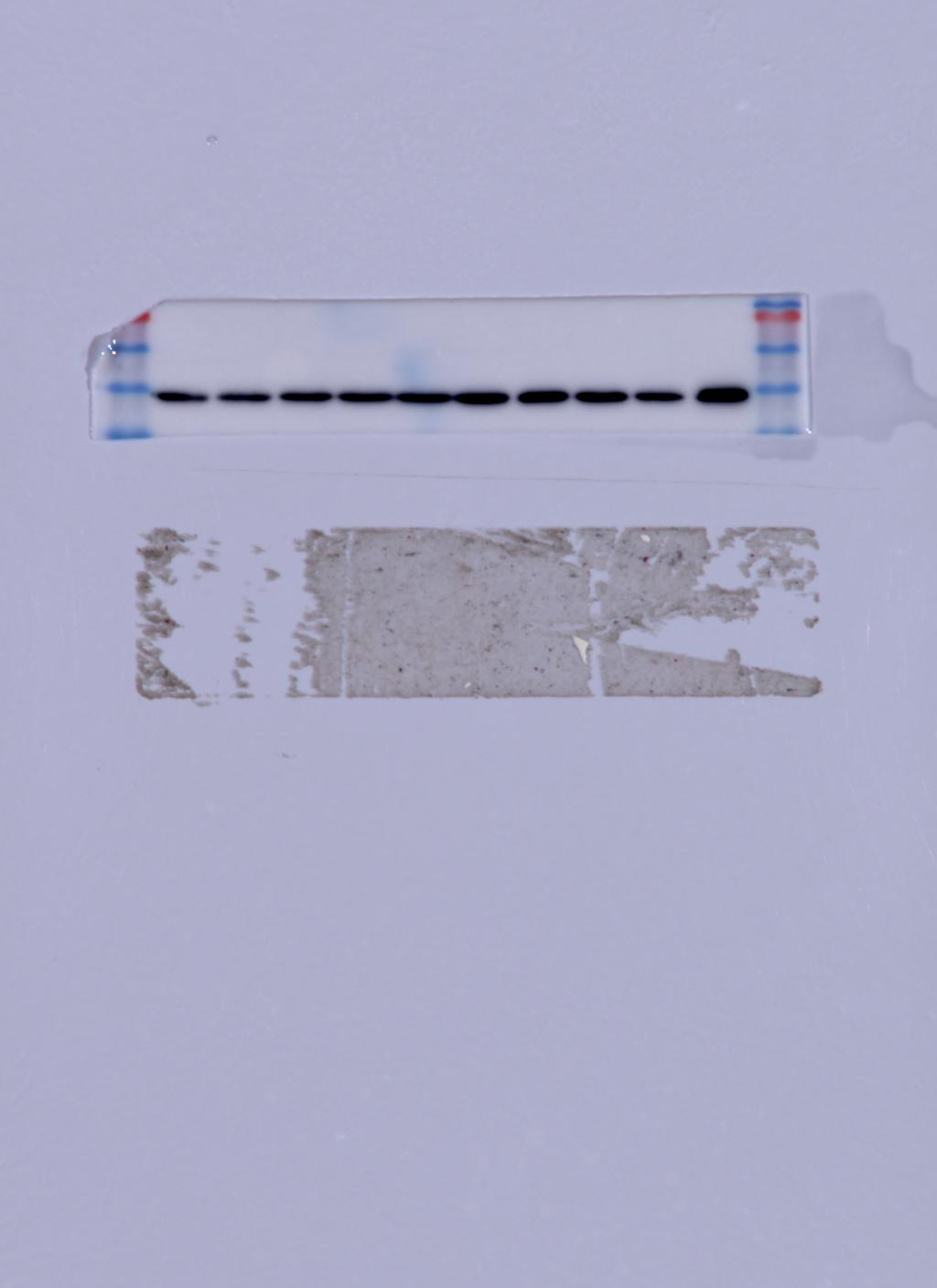


Figure 2 E：Control


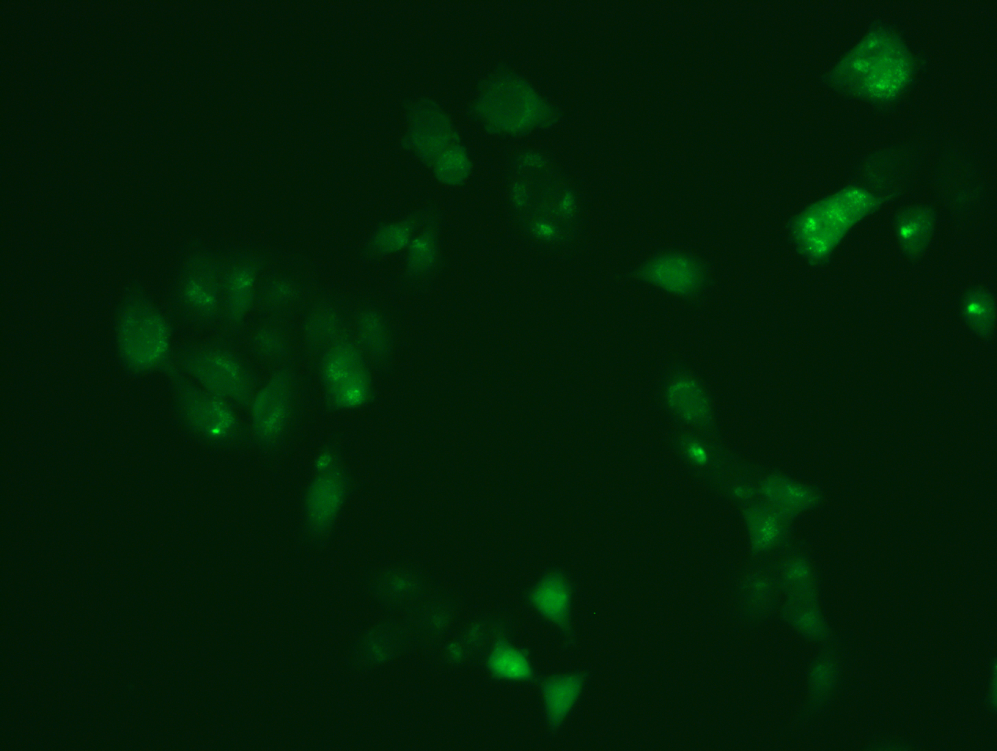


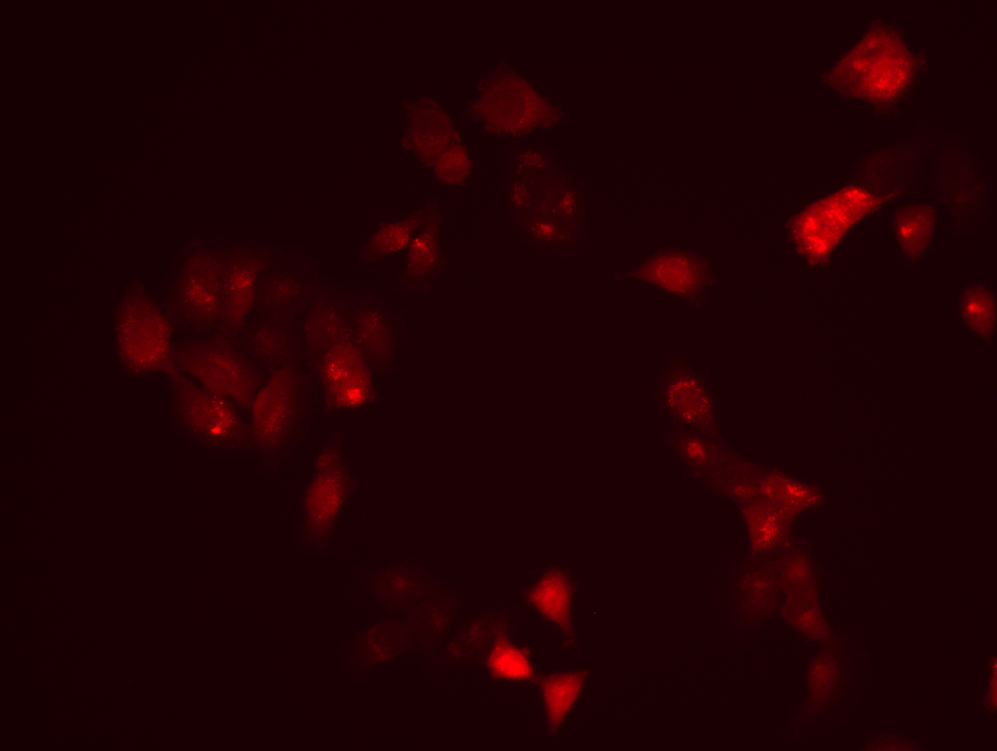

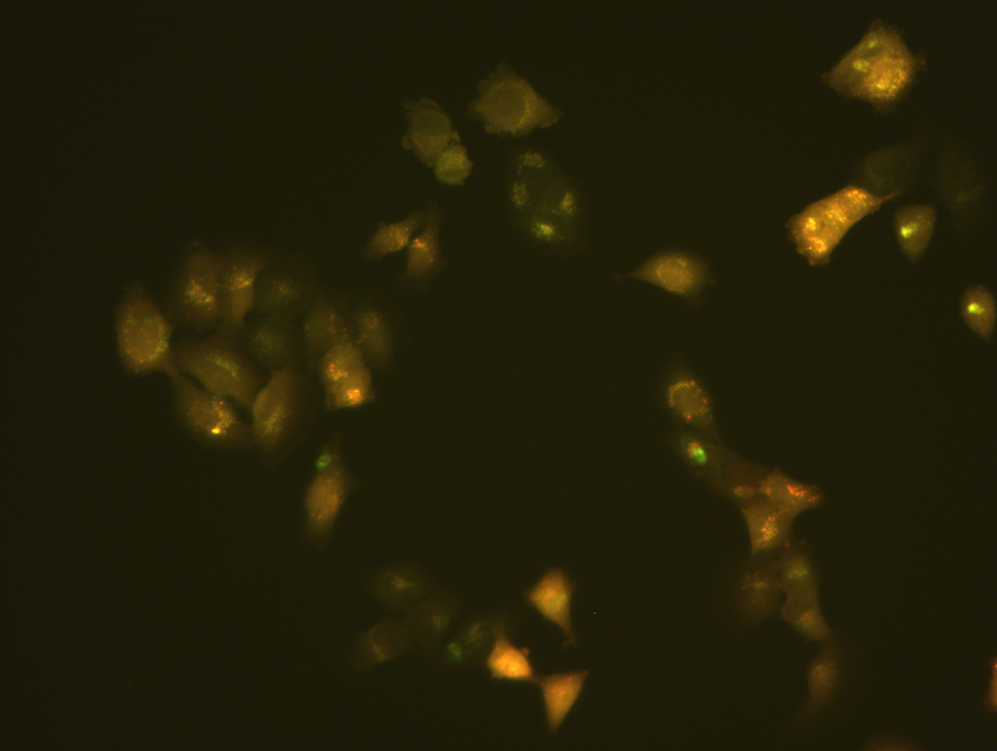


Figure 2 E：ox-LDL


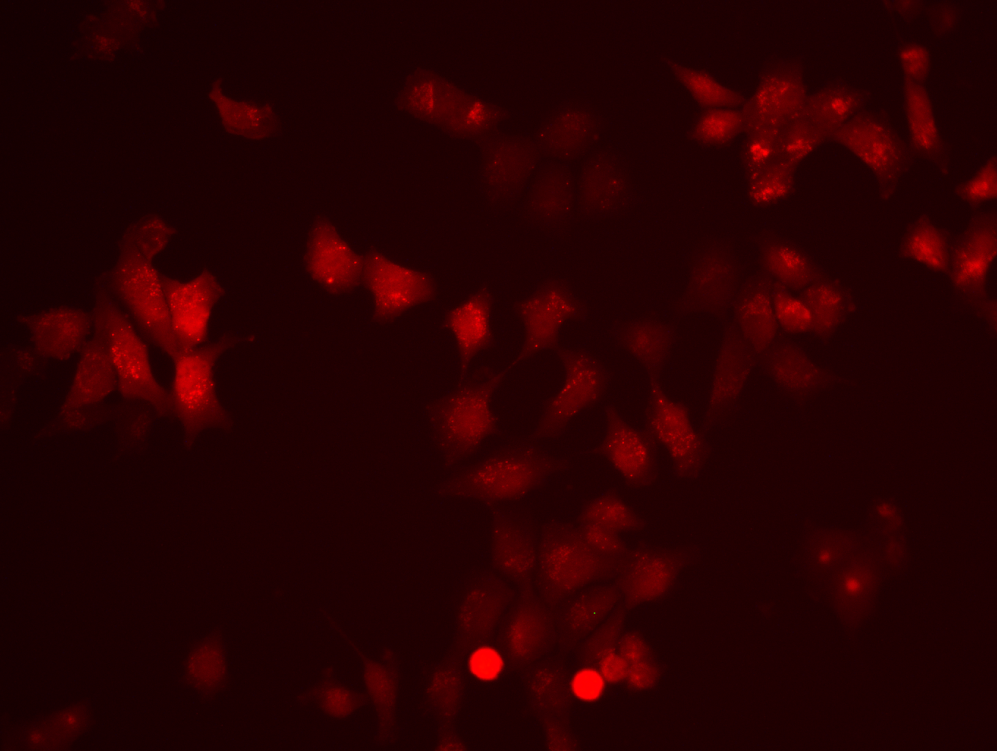


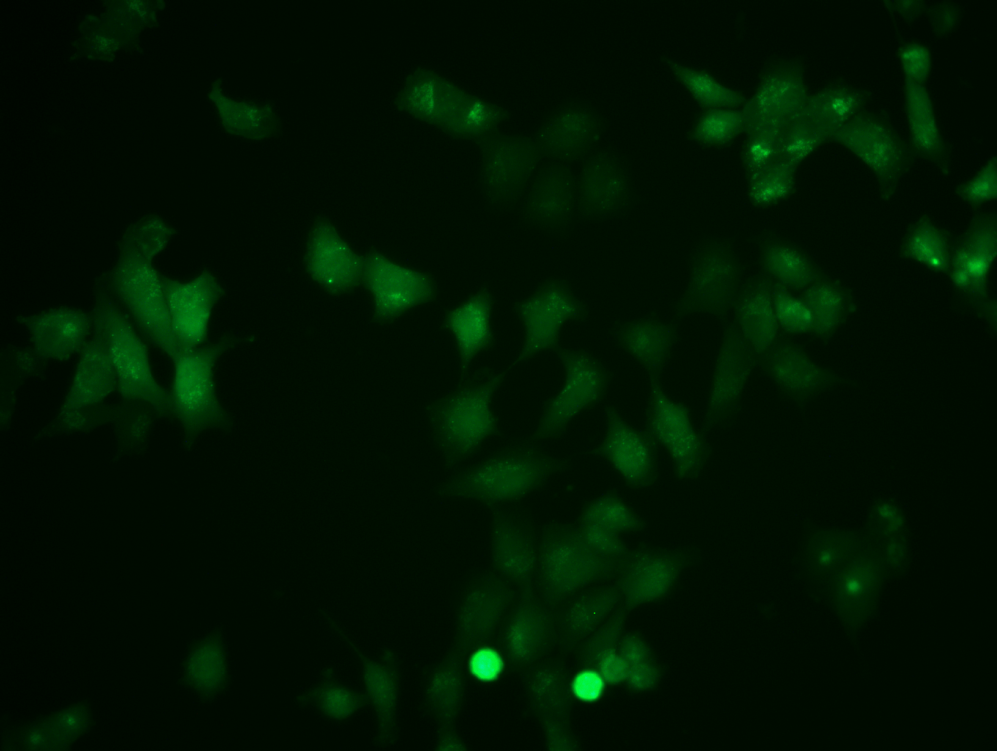

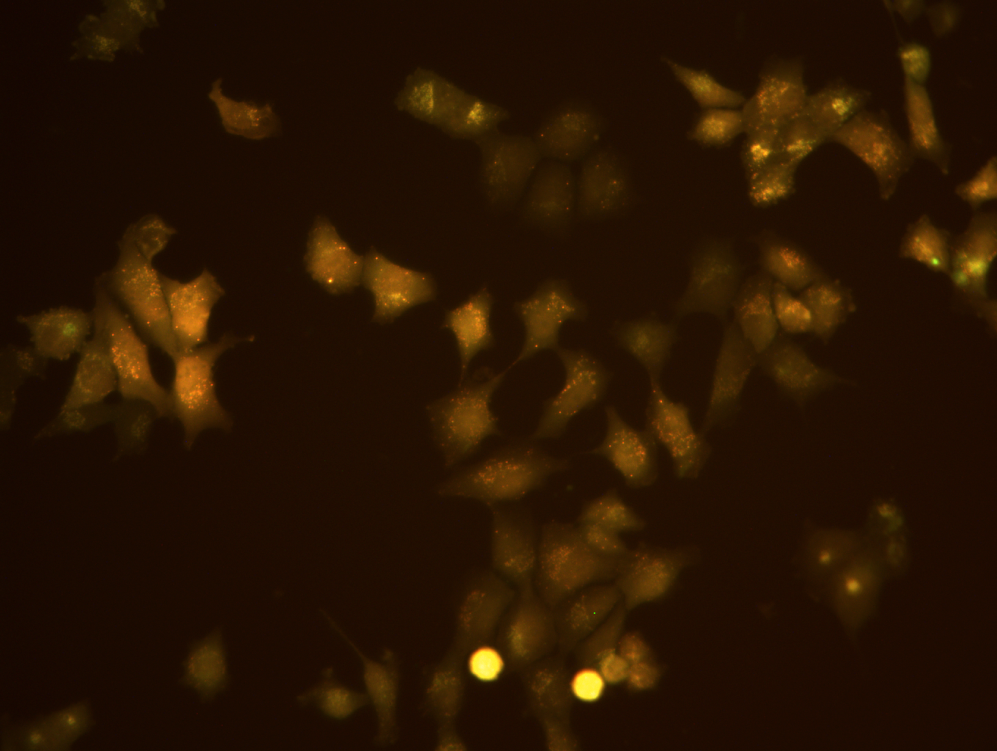


Figure 2 E：Hypoxia


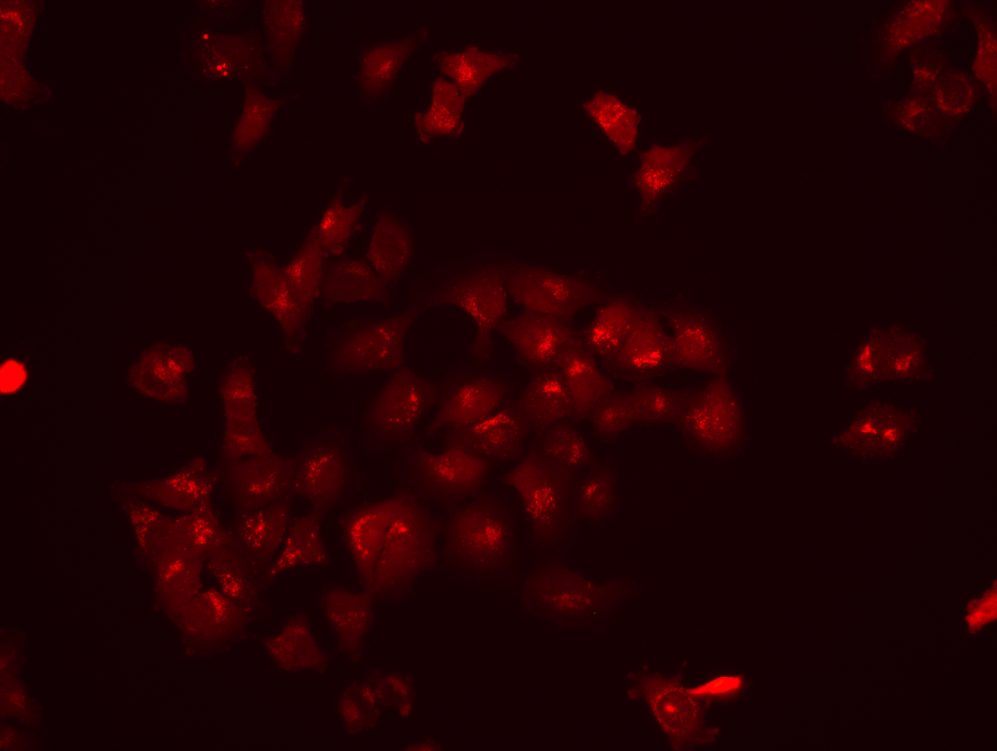


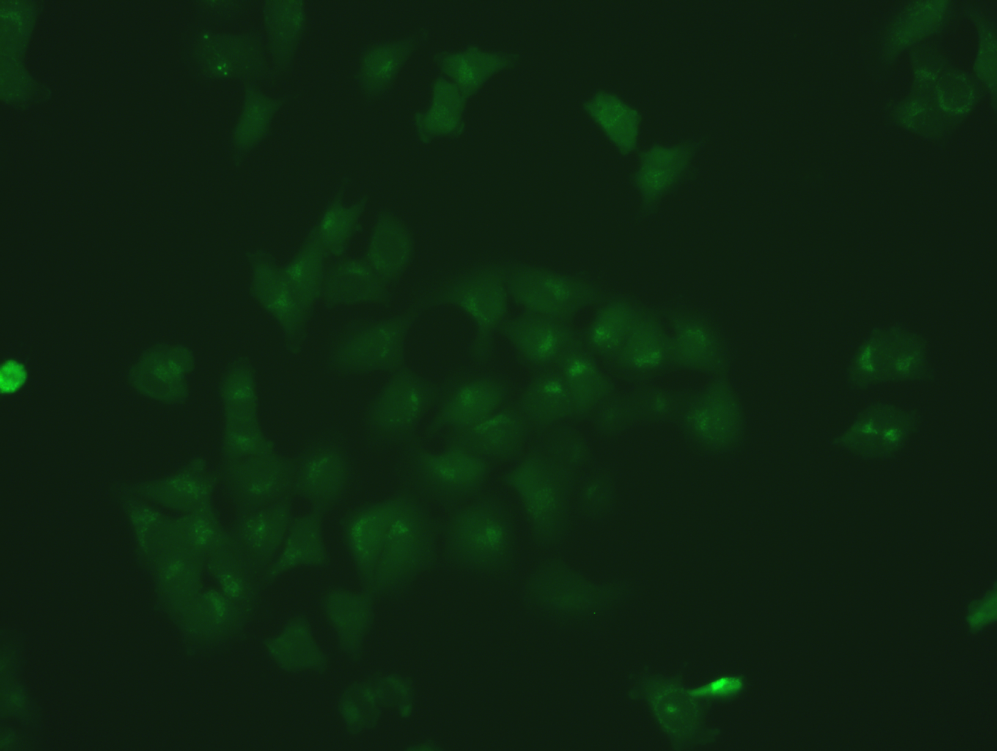

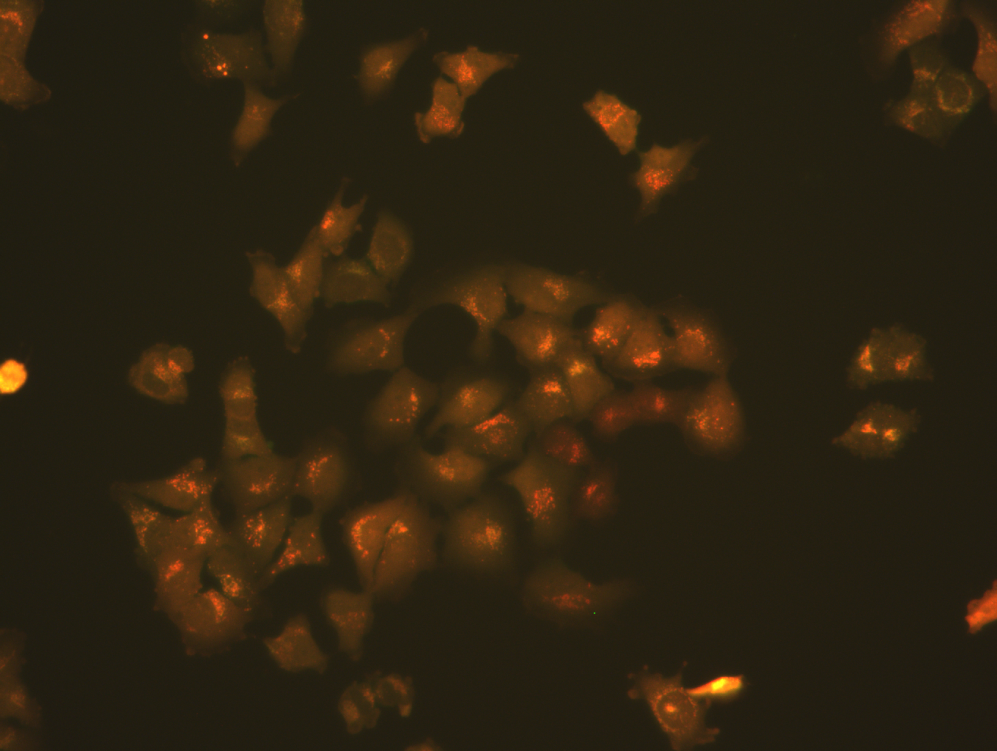


Figure 2 E：Hypoxia+ox-LDL


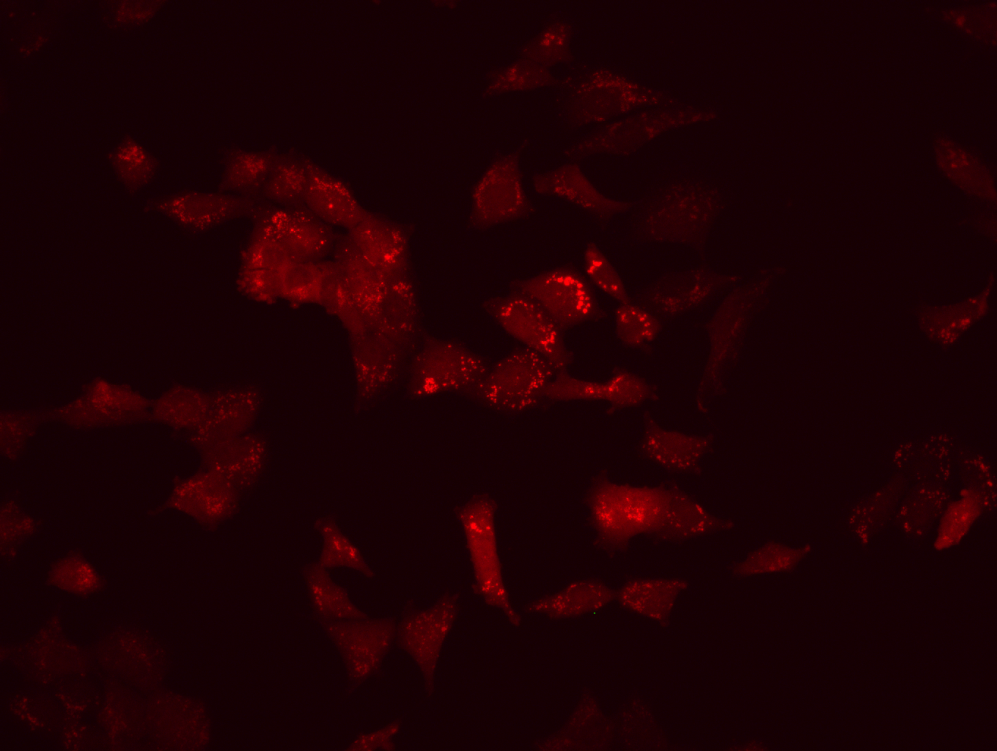


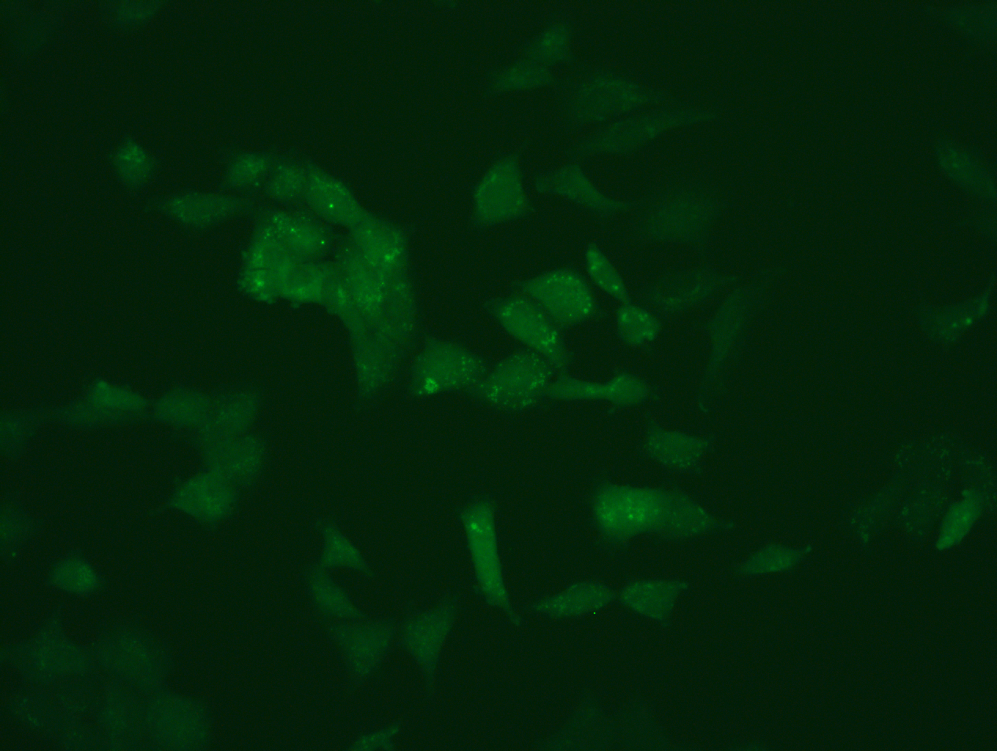

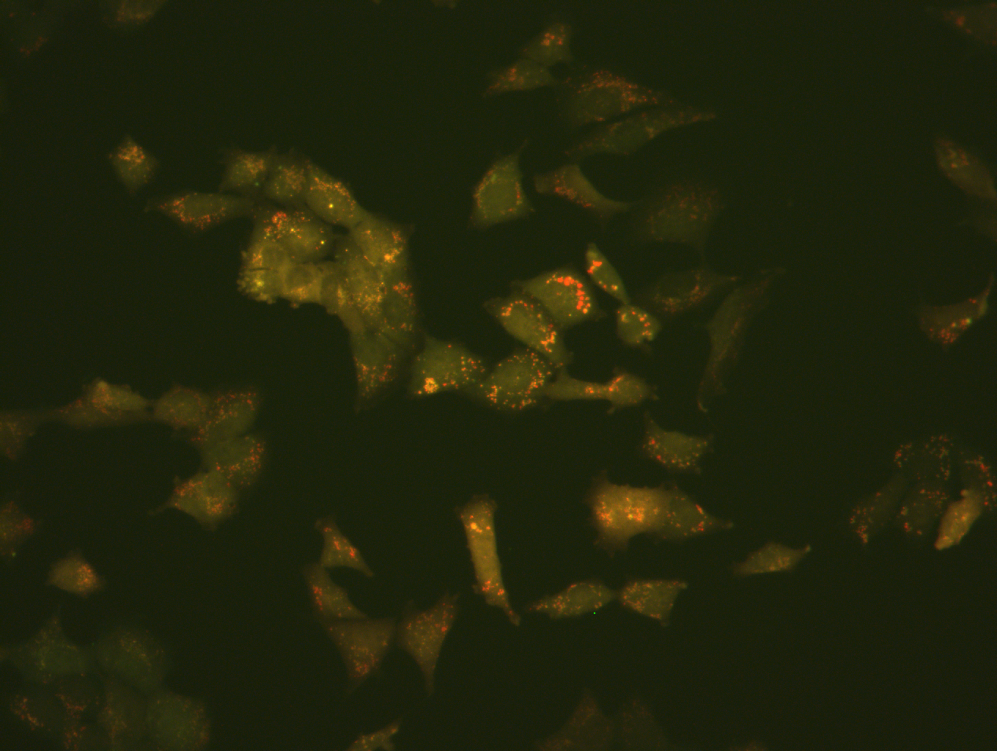


Figure 3 A


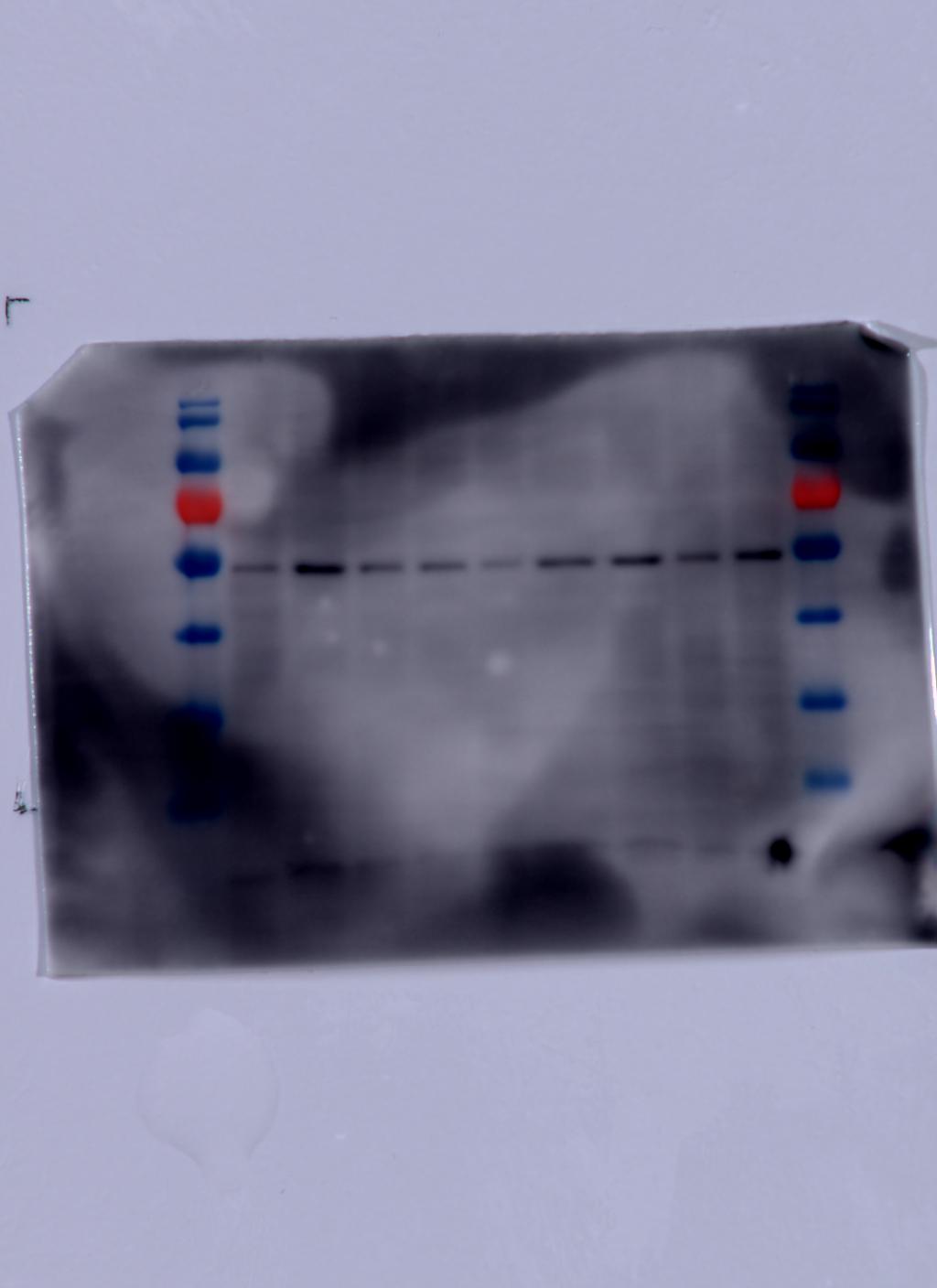


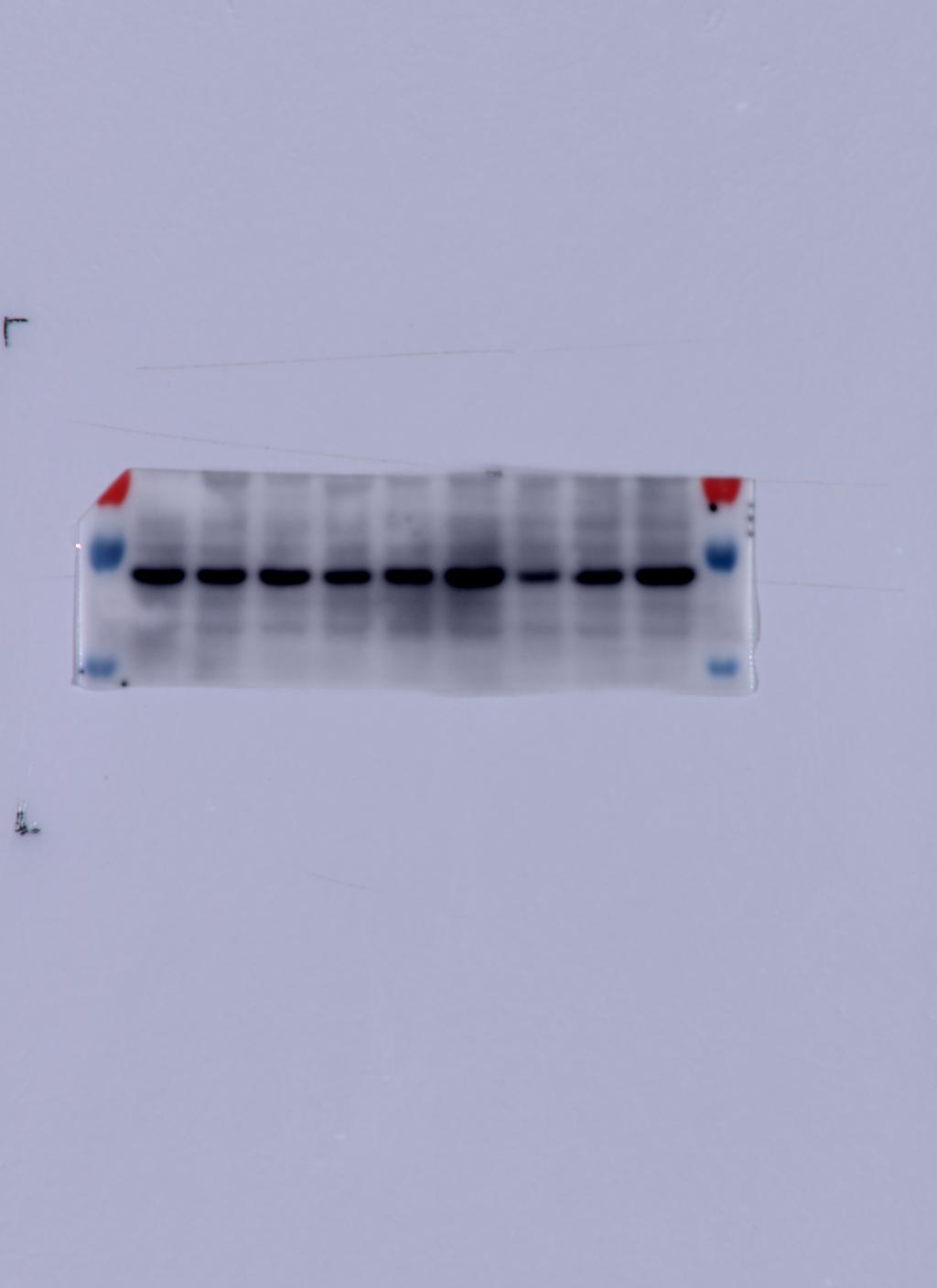


Figure 4 B ：LC3B+β-Tubulin


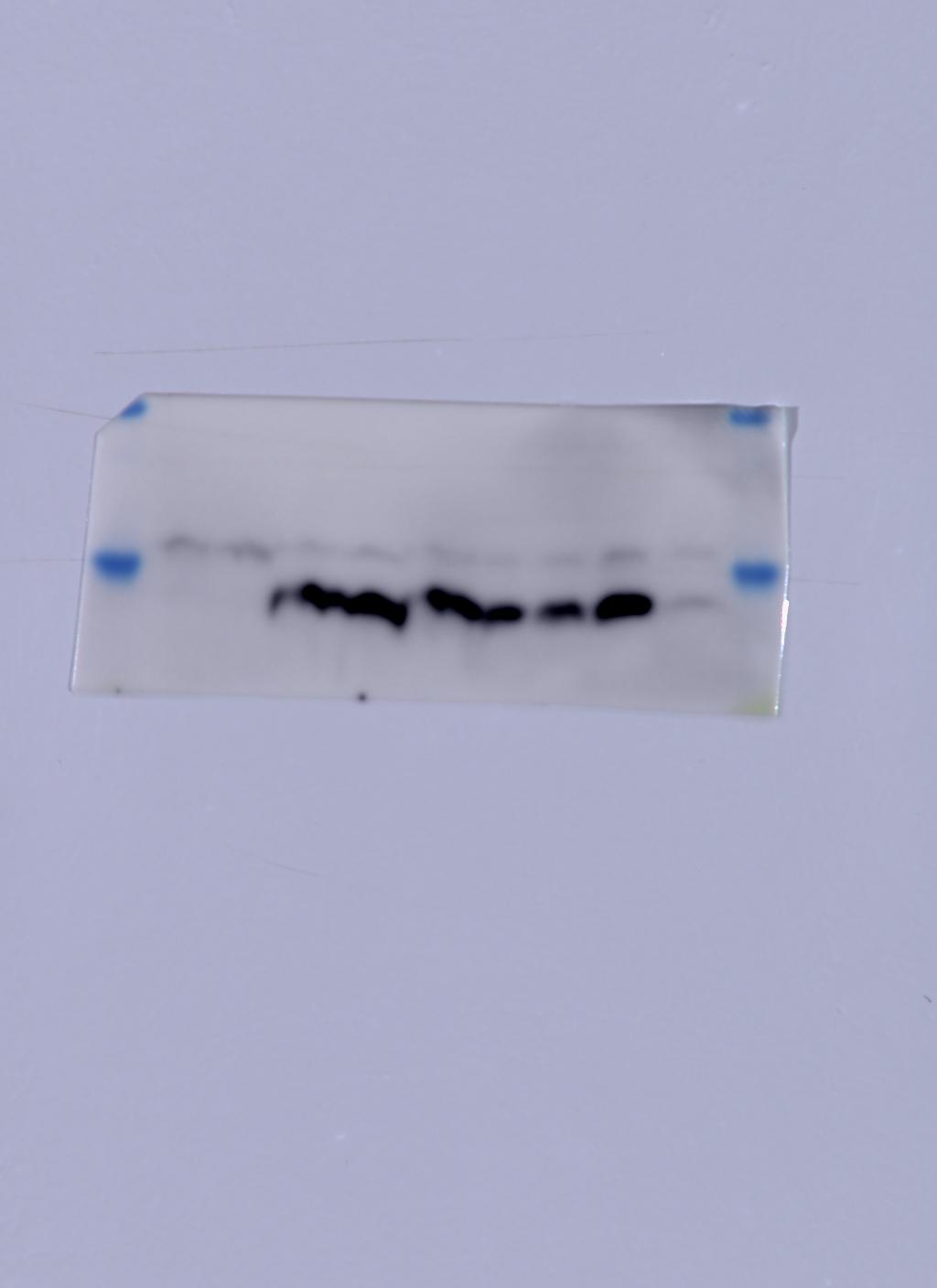

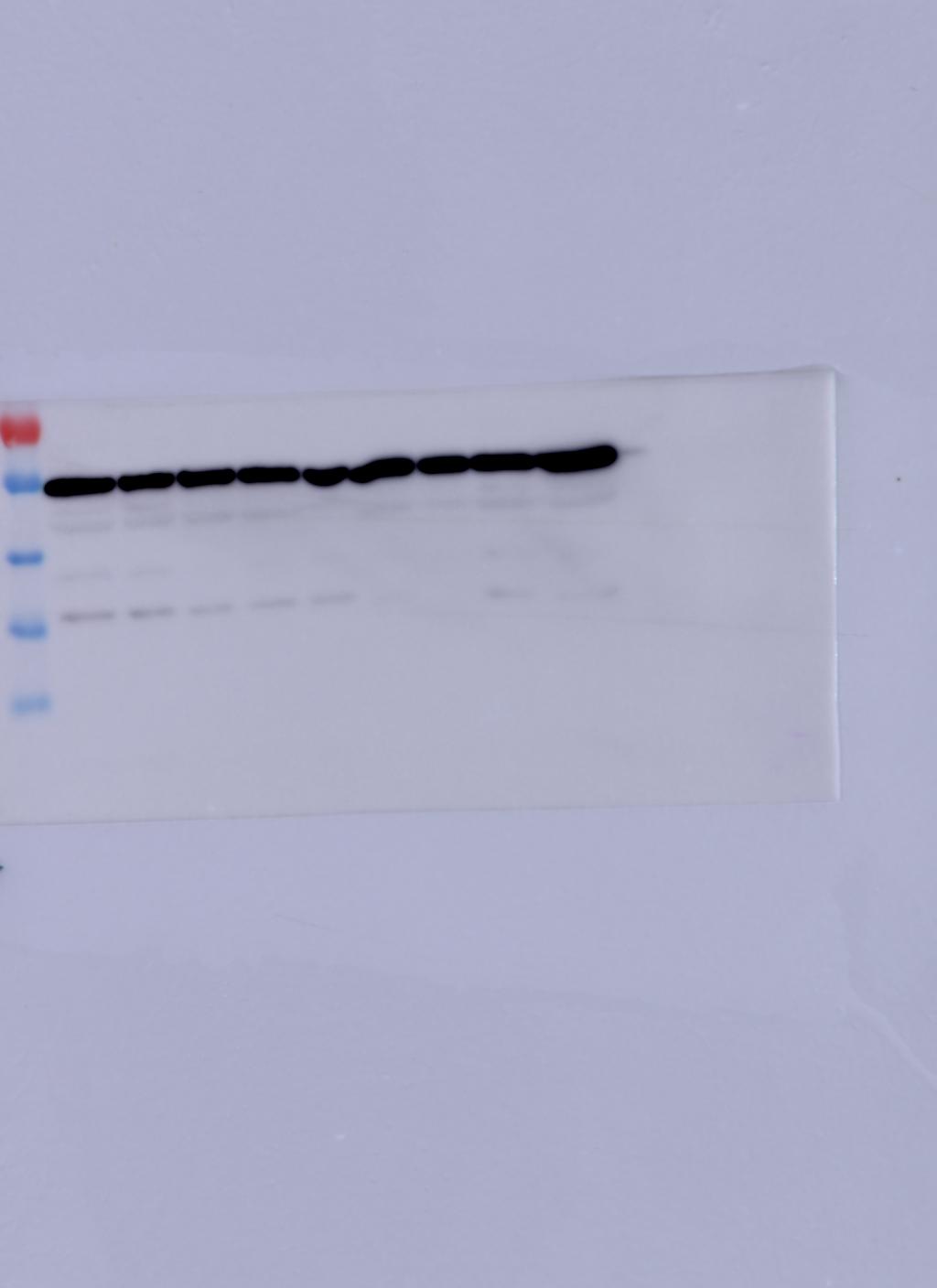


Figure 4 B ：Bax+β-Tubulin


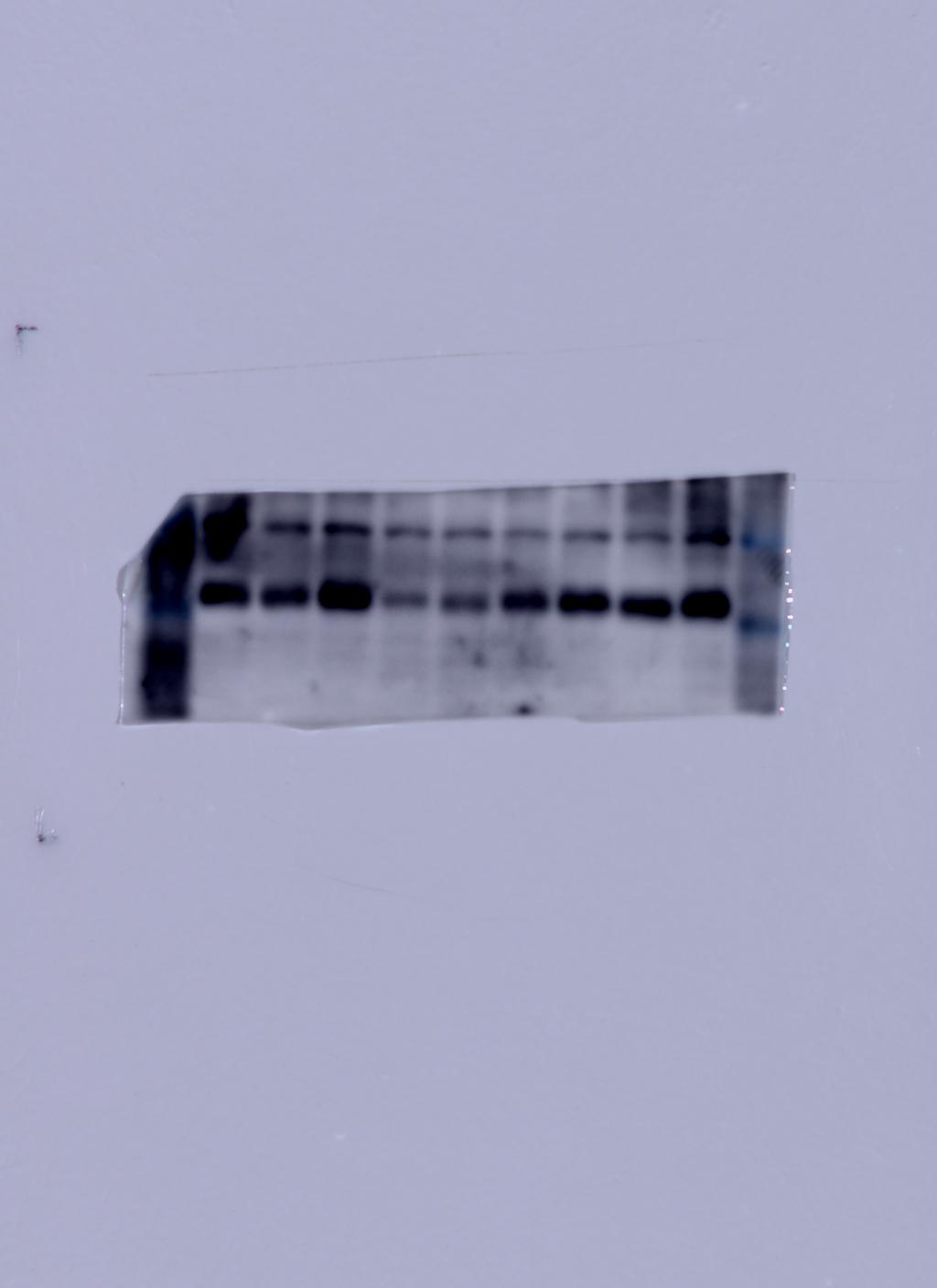

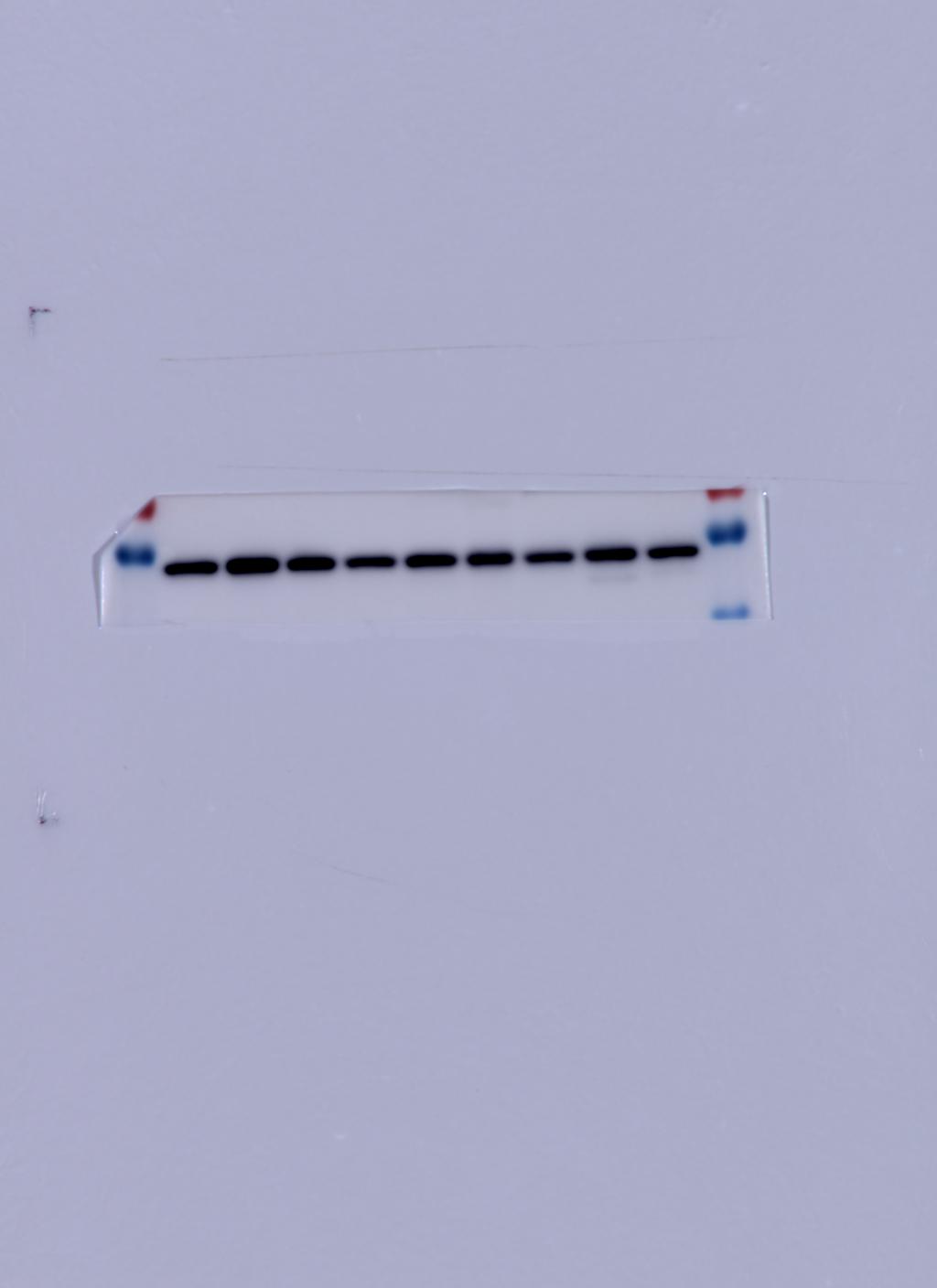


Figure 4 B ：VEGF+β-Tubulin


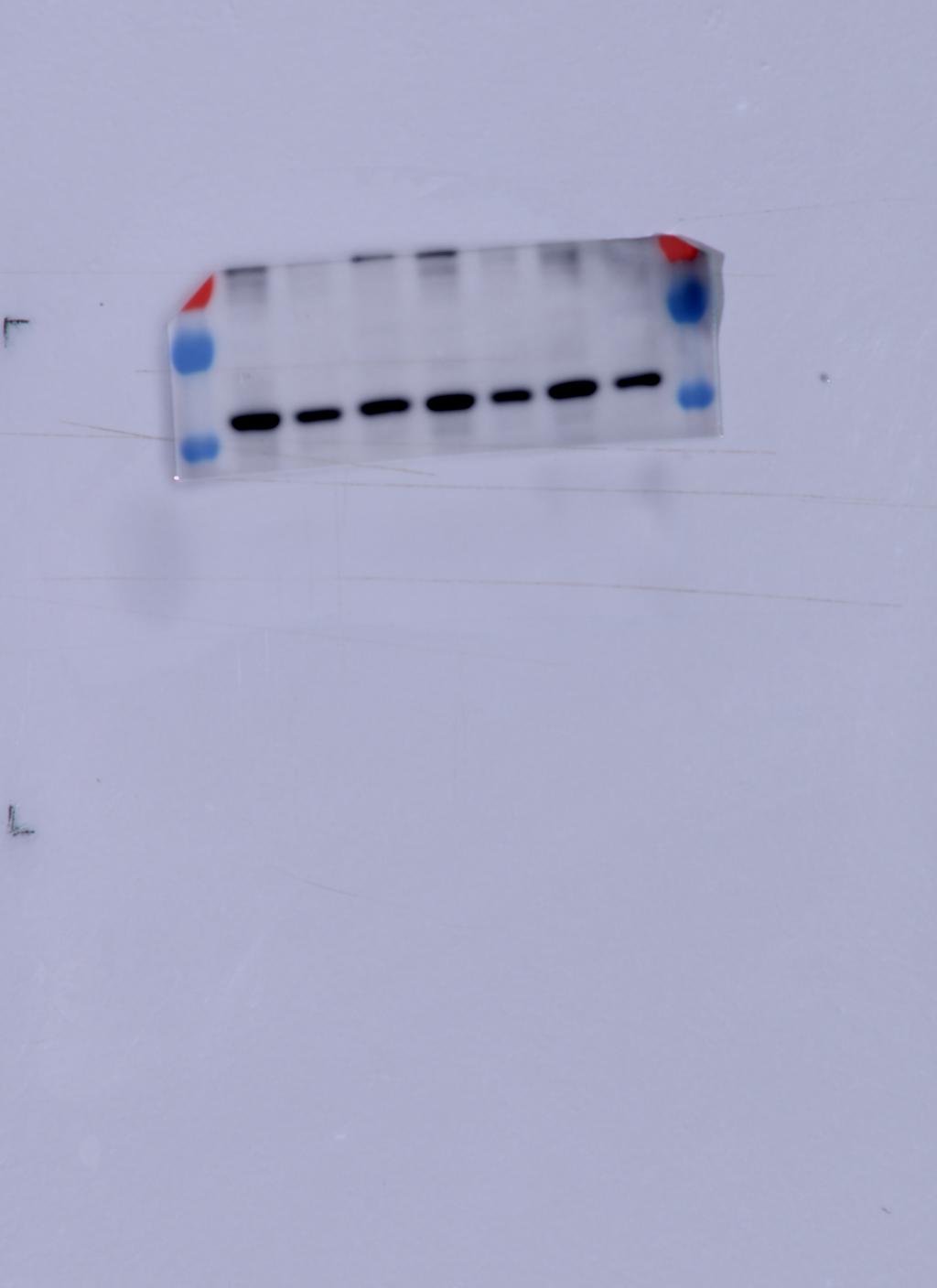

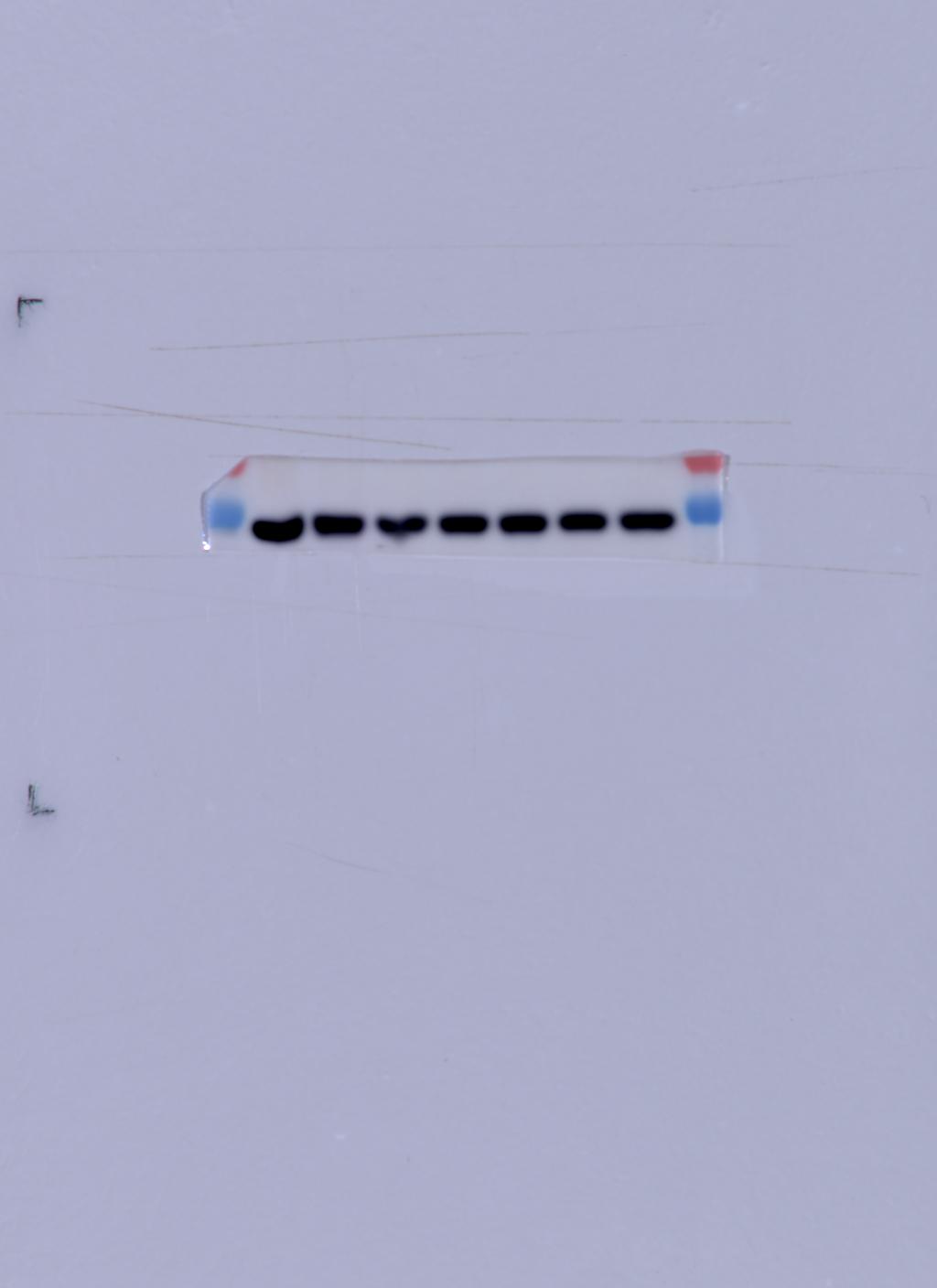


Figure 4 B ：FLT1+GAPDH


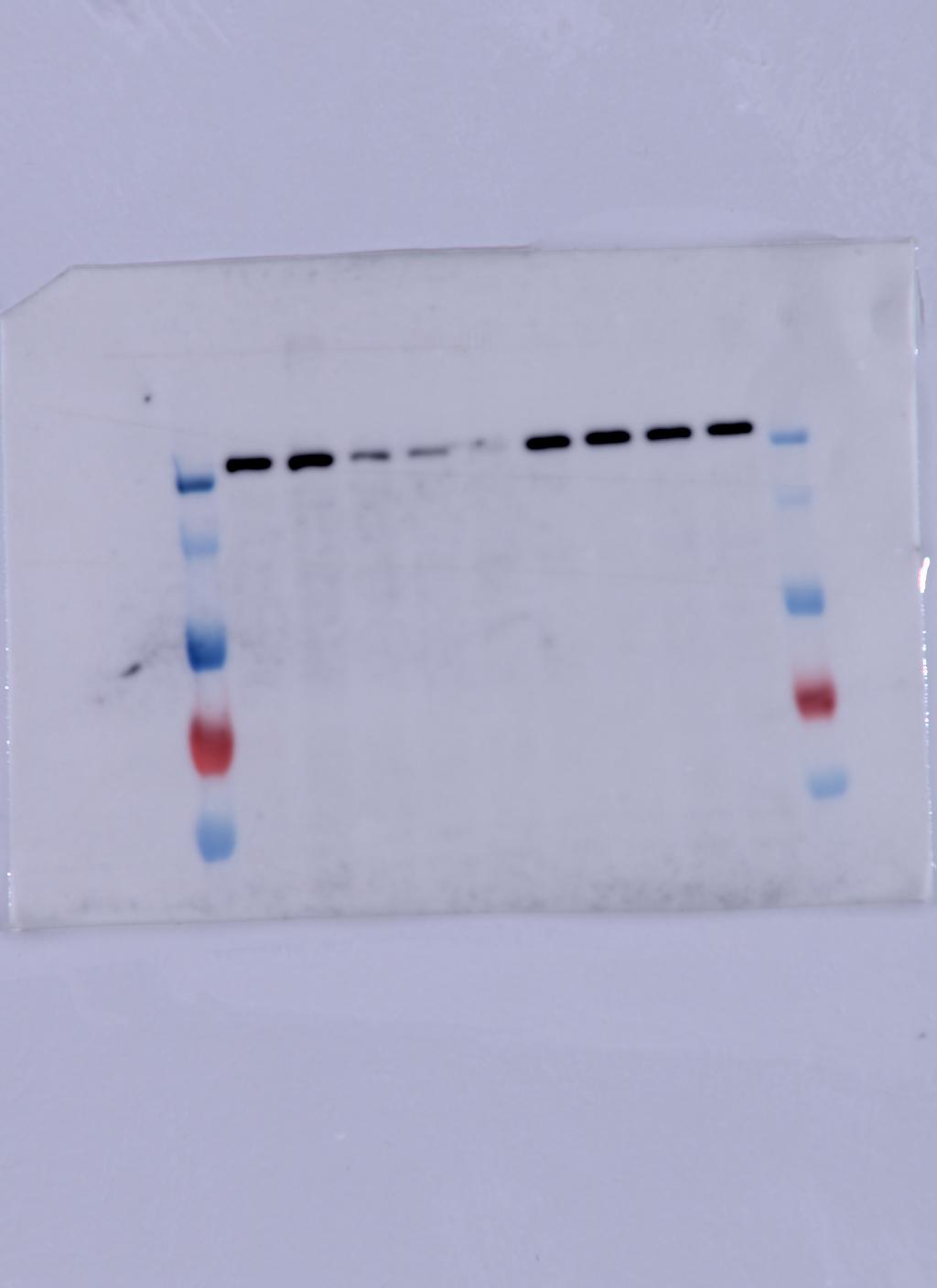

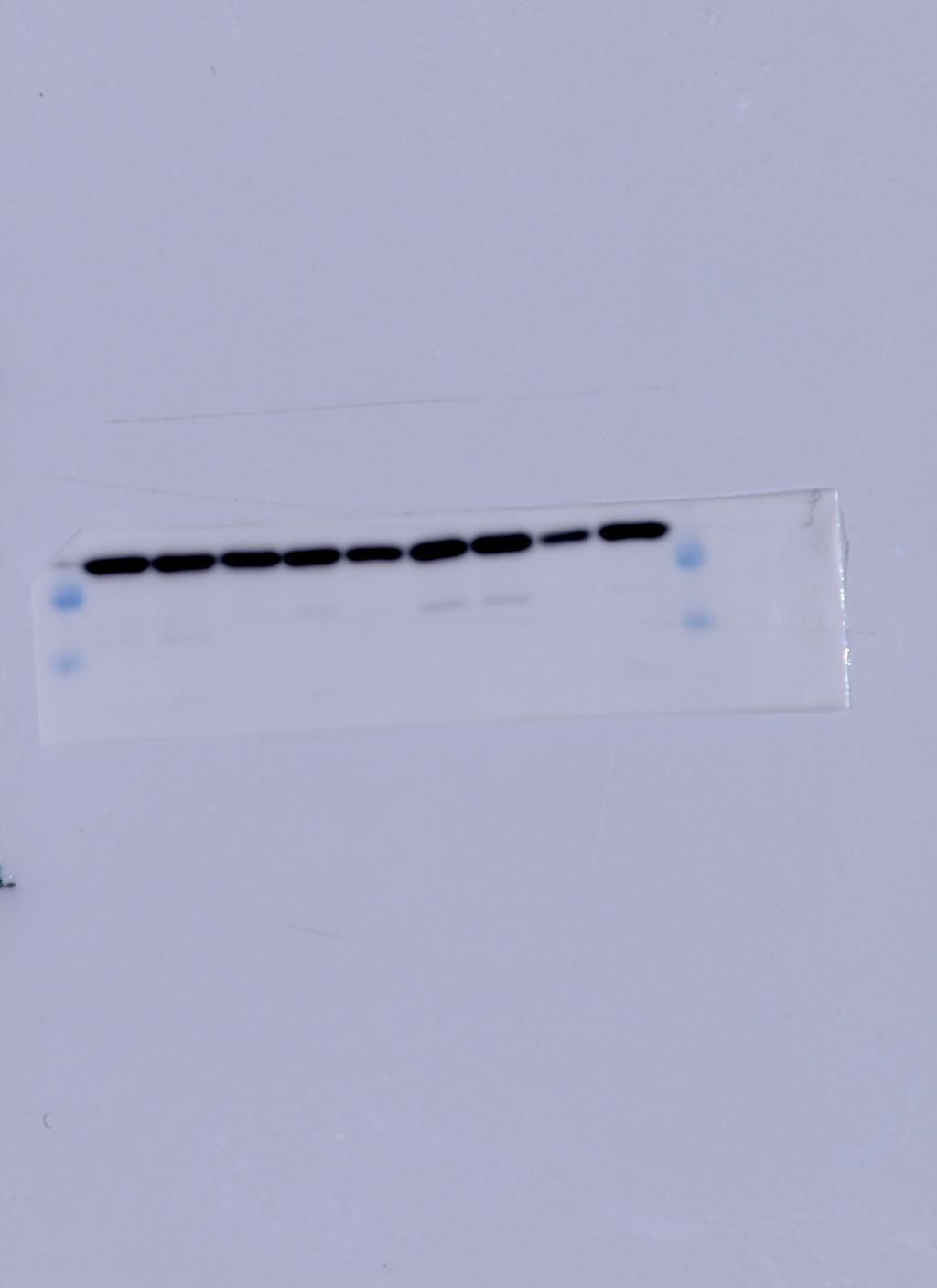

Supplement: Supplementary file 3 [file DataSheet1.DOCX]
